# A splice site-sensing conformational switch in U2AF2 is modulated by U2AF1 and its recurrent myelodysplasia-associated mutation

**DOI:** 10.1093/nar/gkaa293

**Published:** 2020-04-28

**Authors:** Chandani Warnasooriya, Callen F Feeney, Kholiswa M Laird, Dmitri N Ermolenko, Clara L Kielkopf

**Affiliations:** Department of Biochemistry and Biophysics and Center for RNA Biology, University of Rochester School of Medicine and Dentistry, Rochester, NY 14642, USA

## Abstract

An essential heterodimer of the U2AF1 and U2AF2 pre-mRNA splicing factors nucleates spliceosome assembly at polypyrimidine (Py) signals preceding the major class of 3′ splice sites. U2AF1 frequently acquires an S34F-encoding mutation among patients with myelodysplastic syndromes (MDS). The influence of the U2AF1 subunit and its S34F mutation on the U2AF2 conformations remains unknown. Here, we employ single molecule Förster resonance energy transfer (FRET) to determine the influence of wild-type or S34F-substituted U2AF1 on the conformational dynamics of U2AF2 and its splice site RNA complexes. In the absence of RNA, the U2AF1 subunit stabilizes a high FRET value, which by structure-guided mutagenesis corresponds to a closed conformation of the tandem U2AF2 RNA recognition motifs (RRMs). When the U2AF heterodimer is bound to a strong, uridine-rich splice site, U2AF2 switches to a lower FRET value characteristic of an open, side-by-side arrangement of the RRMs. Remarkably, the U2AF heterodimer binds weak, uridine-poor Py tracts as a mixture of closed and open U2AF2 conformations, which are modulated by the S34F mutation. Shifts between open and closed U2AF2 may underlie U2AF1-dependent splicing of degenerate Py tracts and contribute to a subset of S34F-dysregulated splicing events in MDS patients.

## INTRODUCTION

**Figure 1.**
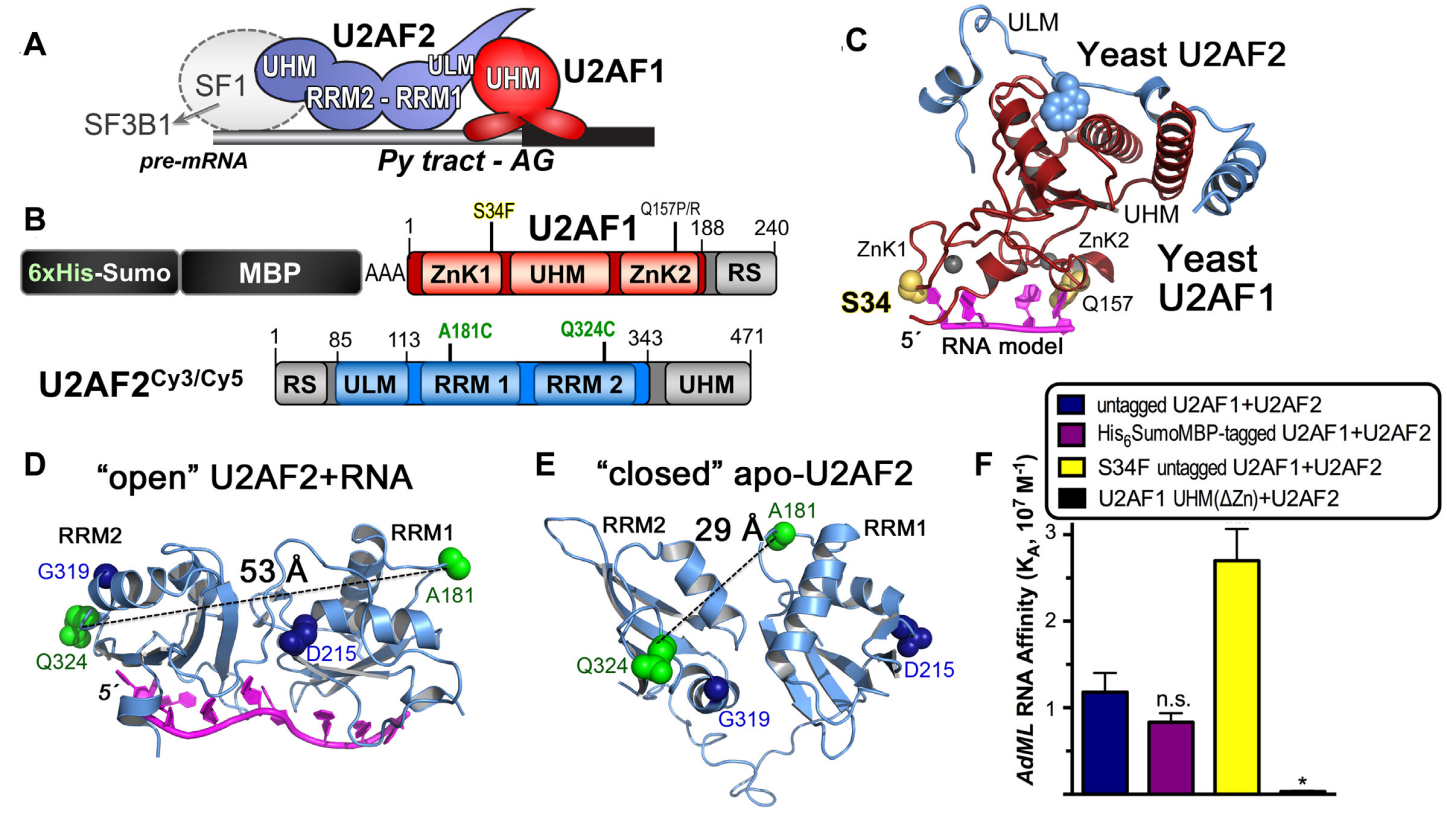
Overview of U2AF1 and U2AF2 domains, structures and labeling strategy. (**A**) U2AF1 and U2AF2 subunits recognize the polypyrimidine (Py) and AG-dinucleotide consensus signals at 3′ splice sites of pre-mRNA transcripts. The SF1 subunit is thought to exchange with SF3B1 during spliceosome assembly. (**B**) U2AF1 and U2AF2 domains and constructs used for smFRET (emphasized by color). The U2AF2 domains include two RNA recognition motifs (RRM1 and RRM2) and a U2AF ligand motif (ULM). U2AF1 domains include zinc knuckles (ZnK1 and ZnK2) and a central U2AF homology motif (UHM) that mediates heterodimerization. The locations of recurrent S34F and Q157P/R cancer-associated U2AF1 mutations are marked. The U2AF1 construct includes a C67S mutation and a C-terminal alanine to enhance protein expression and is an N-terminal fusion with 6xhistidine, sumo, triple-alanine linker, and maltose-binding protein (MBP) tags for surface tethering. (**C**) Structure of fission yeast U2AF1 bound to U2AF2 ULM (PDB ID: 4YH8) modeled with RNA by superposition with PDB ID’s 3D2S and 5GM6 as described (34). Residues corresponding to the MDS-affected residues are yellow spheres. (**D**) The open U2AF2 RRM1-RRM2 conformation bound to poly-uridine oligonucleotide (magenta) (PDB ID 5EV4). (**E**) The closed conformation of apo-U2AF2 (PDB ID 2YH0). For (D, E), the U2AF2 RRMs are labeled at unique cysteine mutations of A181 and Q324 (green spheres); the D215/G319 residues (navy spheres) promote the ‘closed’ conformation following mutation to R. (**F**) Binding affinities of U2AF heterodimer variants for the *AdML* splice site (5′-fluorescein-CUGUCCCUUUUUUUUCAC**AG**|CUCGCGGUUGAG, where | is the spliced junction). Details including the fitted binding curves, construct boundaries and protein preparations are given in Supplementary Figure S1. Not significant (n.s.) *P> 0.05; *P< 0.05; ***P<0.0005 from two-tailed unpaired *t*-tests with Welch's correction of the average values from three experiments for the wild-type proteins and six experiments for the S34F mutant (comparison to untagged heterodimer).

During eukaryotic gene expression, introns are removed from the pre-mRNA transcripts to prepare the mature mRNA for translation. A multicomponent spliceosome machinery of proteins and small nuclear (sn)RNAs assembles in a tightly-controlled, stepwise process on the consensus sequences of pre-mRNA splice sites (1). At the major class of 3′ splice sites, a heterodimer of U2AF1 and U2AF2 (also called U2AF^35^ and U2AF^65^) recognizes an consensus AG-dinucleotide at the splice site junction and a preceding polypyrimidine (Py) tract (Figure 1A). This ribonucleoprotein complex recruits the U2 small nuclear ribonucleoprotein particle (snRNP) of the spliceosome. Whereas U2AF1 has a specific role in splicing so-called AG-dependent splice sites that lack a clear Py tract signal (2–4), the U2AF2 subunit serves general functions for splicing of uridine-rich sites (5). These different U2AF1-dependencies and degeneracy of the Py tract signal are thought to regulate alternative splicing of multi-exon genes, which generates numerous transcript variants in multicellular eukaryotes (6). Beyond their traditional role as pre-mRNA splicing factors, the U2AF1 and U2AF2 subunits also function in translational repression and 3′ end processing (7–12).

Acquired mutations of pre-mRNA splicing factors are a prevalent source of splice site dysregulation among hematologic malignancies and myelodysplastic syndromes (MDS) (13). U2AF1 is one of the recurrently mutated splicing factors in MDS, alongside SF3B1, SRSF2 and ZRSR2. These affected splicing factors synergize for early spliceosome assembly. Interestingly, ZRSR2 is a U2AF1-paralogue of the minor, U12-type spliceosome (14,15), whereas SRSF2 shares U2AF1/U2AF2 functions of annealing pre-mRNA consensus elements with spliceosomal RNAs, such as the U2 snRNA (16–18). Protein-protein interactions between SF3B1 and U2AF2 are believed to assist U2 snRNP association with the 3′ splice site (19,20). The cancer-associated mutations of pre-mRNA splicing factors cluster at hotspots and typically result in the same amino acid substitutions. Most commonly, U2AF1 acquires an S34F-encoding mutation (∼80% of MDS-associated *U2AF1* mutations), or in a few cases, S34Y or Q157P/R mutations (21–24). The affected S34 and Q157 residues of U2AF1 are located at the putative RNA interfaces of two zinc knuckle motifs (Figure 1B, C and described below). The S34F mutation subtly modulates U2AF1–RNA binding and splicing in a manner that correlates with the identity of the nucleotide preceding the AG-dinucleotide at the 3′ splice site (25–27). The S34F mutation also alters non-splicing functions of U2AF1, including translational repression (7) and alternative polyadenylation (28). Less frequently, the U2AF2 subunit acquires cancer-associated missense mutations, which tend to cluster at the U2AF2/RNA or inter-domain interfaces (29).

Breakthrough studies have revealed near-atomic resolution structures of spliceosome assemblies (reviewed in (30)). However, gaps remain among the currently available views of the earliest stage of splice site recognition and spliceosome recruitment. Structures have been determined for portions of the U2AF1 and U2AF2 subunits (Figure 1C-E). A ‘U2AF Homology Motif’ (UHM) heterodimerization domain of U2AF1 binds an N-terminal ‘U2AF Ligand Motif’ (ULM) of U2AF2 (31,32). The MDS-relevant zinc knuckles of U2AF1 fold on the UHM surface in the 3D structure of the fission yeast U2AF1 homologue bound to the U2AF2 ULM (33). Models of the U2AF1–splice site RNA complex (such as shown in Figure 1C) and complementary RNA binding experiments suggest that the U2AF1 zinc knuckle motifs comprise the major AG-recognition interface, and also that the MDS mutation-prone S34 and Q157 residues are located near the RNA interface (26,33,34). For splice site recognition by U2AF2, two tandem RNA recognition motifs (RRM1 and RRM2) bind the Py tract splice site signal, and numerous structures have been determined of this U2AF2 region. Initial crystal structures of a U2AF2 RRM1/RRM2 fragment detailed the local RRM/Py tract interactions, but left the native RRM arrangement unknown (35–38). Pivotal nuclear magnetic resonance/paramagnetic relaxation enhancement (NMR/PRE) and crystal structures of the intact, U2AF2 RRM-containing region revealed an open, side-by-side arrangement of the U2AF2 RRMs bound to strong Py tracts comprising contiguous uridines (39,40) (Figure 1D). Moreover, in the absence of RNA, an NMR/PRE approach characterized a major closed conformation of U2AF2, which masks the typical RNA binding surface of RRM1 and leaves only the uridine-stringent RRM2 available for RNA binding (40) (Figure 1E).

In addition to X-ray crystallography and NMR/PRE, the complementary methods of small-angle X-ray scattering (SAXS) and single molecule Förster resonance energy transfer (smFRET) have provided further insights into U2AF2 conformational dynamics. Small-angle X-ray scattering (SAXS) studies of the U2AF2 RRM-containing region suggests that an ensemble of associated and detached U2AF2 RRMs accompany the closed conformation in the absence of RNA ligand (41–43). The conformational dynamics of the U2AF2 RRM1/RRM2 region has been studied by smFRET in the absence and presence of RNA sites (39,44) and also as a complex with the UHM heterodimerization domain of U2AF1 (44). The RRM1/RRM2 region of the isolated U2AF2 subunit shows transitions in the RNA-free state, in agreement with the loose association of the U2AF2 RRMs observed using SAXS. Addition of a strong, uridine-tract RNA increases a population of FRET values consistent with the open, RNA-bound conformation of U2AF2 (39,44), and U2AF2 dynamics increased when adenosine-tracts interrupted the uridines (44). Addition of the minimal UHM heterodimerization domain of U2AF1, which the remaining core following truncation of the flanking zinc knuckles, also increased the population of open U2AF2 conformations. Yet, the minimal U2AF1 UHM lacked the sites of MDS-associated mutations, which limited the smFRET investigations to relatively rare, cancer-associated mutations of U2AF2 (L187V and M144I) (44). At present, the influences on the U2AF2 conformational ensemble of natural splice site sequences, the U2AF1 zinc knuckle—RNA interface, or the prevalent U2AF1 MDS-associated mutations remains unknown.

In a step toward addressing this question, we prepared heterodimers of U2AF2 bound to an extended U2AF1 construct containing the zinc knuckle regions, and used smFRET to characterize the influence of these U2AF1 zinc knuckles, the cancer-associated S34F mutation, and a variety of representative 3′ splice site RNAs on the U2AF2 inter-RRM dynamics. Heterodimerization with U2AF1 appeared to stabilize the U2AF2 conformations in a sufficient time frame for the initiation of pre-mRNA splicing. In the absence of RNA, U2AF1 stabilizes a U2AF2 conformation with high apparent FRET efficiency, which by site-directed mutagenesis, corresponds on average to the closed inter-RRM conformation. A strong, uridine-rich splice site switches U2AF2 to a lower FRET state that is likely to correspond to the open conformation. Remarkably, a significant population of the U2AF2 conformations remains in the closed state for complexes with weak, uridine-poor splice sites of gene transcripts that depend on U2AF1 for efficient splicing or are dysregulated by the S34F mutation. This conformational difference could contribute to a conditional requirement for U2AF1 to promote splicing of a degenerate class of splice sites. The U2AF1 S34F-mutant alters the distribution of U2AF2 conformations for a subset of the weak splice site complexes, which suggests a new structural contributor to dysregulated gene expression in MDS.

## MATERIALS AND METHODS

### Protein expression and purification

All proteins were expressed in BL21(DE3) (for expression or co-expression of pCDF-1b vectors) or BL21 (for pGEX-6p vectors) by overnight induction with 0.2 mM isopropyl β-d-1-thiogalactopyranoside at 18°C. In general, affinity purifications followed the HiTrap manufacturer's protocols and included cOmplete™ EDTA-free protease inhibitors (Millipore-Sigma), 6 mM β-mercapto-ethanol, and 0.2 tris(2-carboxyethyl) phosphine (TCEP) reducing agents in the buffers. The final size exclusion chromatography (SEC) buffers comprised 150 mM NaCl, 25 mM HEPES pH 7.0, 20 μM ZnCl_2_, 3% Glycerol, 0.5 mM TCEP, 0.5 mM DTT. The S34F mutation was introduced into the corresponding wild-type U2AF1 constructs and the S34F U2AF1 protein showed no detectable different in solubility or yield compared to the unmodified parent. Additional details are described below.

For FRET experiments with the heterodimer, the wild-type and S34F-mutant U2AF1 boundaries included residues 1–188 of Refseq NP_006749. A serine was substituted for a poorly conserved cysteine (C67S) as described (32) and a C-terminal alanine was added to enhance soluble protein expression. The U2AF1 construct was fused at the N-terminus with a three-alanine linker to His_6_-Sumo-maltose binding protein (MBP) tag in a modified pCDF-1b vector. The U2AF2 subunit included residues 85–342 of NCBI RefSeq NP_001012496 (the predominant isoform in human tissue samples (45)) fused at the N-terminus with a PreScission protease cleavable glutathione-S-transferase (GST) tag using the pGEX6p-1 vector. The U2AF2 boundaries excluded the RS domain and a cysteine-rich C-terminal domain that binds a branch site recognition factor, SF1. The endogenous U2AF2 cysteine was replaced with a natural alanine variation (C305A), and single A181C and Q324C mutations for fluorophore attachment were introduced at locations intended to maximize FRET differences between the open and closed U2AF2 conformations as described (39). The His_6_SumoMBP-U2AF1 and GST-U2AF2 proteins were expressed separately in *Escherichia coli* and purified respectively by HisTrap™ (GE Healthcare, Inc.) and GST-affinity chromatography. Prior to mixing the subunits, the His_6_SumoMBP-U2AF1 protein was further purified by Superdex-75 size exclusion chromatography (SEC), whereas the GST tag was cleaved and separated from U2AF2 by a heparin-affinity chromatography. The S34F mutant U2AF1 and D215R-D319R-mutant U2AF2 were prepared by identical methods as the wild-type parents. The U2AF2 subunit was labeled with fluorophores as described below before reconstitution with the U2AF1 subunit.

For FRET experiments with the isolated U2AF2 subunit, U2AF2 (residues 113–342) was prepared with His_6_ and T7 tags as described (39). The U2AF1-binding site (ULM, residues 85–112) was excluded from this U2AF2 construct to avoid nonspecific binding to the surface.

The constructs encoding the heterodimer of untagged U2AF1 and U2AF2 used for the RNA binding experiments in Figure 1F and Supplementary Figure S1 were described previously (25,27). U2AF2 (residues 85 to the C-terminus of the protein) was expressed as an N-terminal GST fusion from a pGEX-6p vector. The cleavable U2AF1 construct (residues 1–193) was expressed from a modified pCDF-1b vector as an N-terminal fusion with an MBP tag followed by a TEV protease site and GSGGGGS linker. This glycine-rich linker was necessary for efficient cleavage of the MBP tag from the co-expressed heterodimer. The uncleaved, MBP-fused U2AF1 construct was identical to the U2AF1 construct used for FRET. To prepare the heterodimers, the U2AF2 plasmid was co-transformed with plasmids encoding either the MBP-fused or cleavable U2AF1. The heterodimers were purified by sequential MBP-affinity and GST-affinity chromatography. The GST tag was cleaved from U2AF2 by PreScission protease during overnight dialysis into the SEC buffer and removed subtractive GST affinity chromatography. Minor aggregates and remaining GST were separated from the heterodimer by Superdex-200 SEC. To prepare untagged U2AF1–U2AF2, the MBP was first cleaved from the U2AF1 subunit during overnight dialysis with TEV protease before SEC. Mild precipitate was pelleted by centrifugation and the supernatant was further purified by Superdex-200 SEC to separate the untagged heterodimers from TEV and cleaved MBP.

The U2AF1 UHM (ΔZn, residues 43–146 with C67S), which lacks the N- and C-terminal zinc knuckles compared to the U2AF1 construct, was expressed separately from U2AF2 from a modified pGEX-4T vector as a GST-fusion protein with a TEV cleavage site to remove the tag. The U2AF1 UHM was first purified by GST affinity. The GST tag was cleaved from the U2AF1 UHM during overnight dialysis with TEV protease and then removed by anion exchange chromatography. Lastly, the U2AF1 UHM–U2AF2 heterodimer was isolated by SEC of the U2AF1 UHM mixed in slight excess with the pre-purified U2AF2 subunit.

The RNA oligonucleotides (sequences in Supplementary Table S1) were purchased in the deprotected, desalted and HPLC-purified form from Dharmacon, Inc. The RNAs were resuspended at >1 mM concentrations in water and diluted into the indicated buffers for each experiment.

### Sample labeling for smFRET

To remove sulfhydryl reducing agents prior to labeling, the U2AF2 subunit was dialyzed into 500 mM NaCl, 25 mM HEPES pH 7.0, 3% (v/v) glycerol, 0.2 mM TCEP following dilution into dialysis buffer with the addition of 0.01% (w/v) octaethylene glycol monododecyl ether (also called Nikkol, Sigma-Aldrich). The cyanine (Cy)3-maleimide (Combinix, Inc.) and Cy5-maleimide were resuspended in DMSO and mixed in an equimolar ratio immediately prior to use, then added at a 20:1 molar ratio of each dye to U2AF2 protein. The labeling reaction was incubated in the dark at room temperature for two hours and then quenched by addition of 10 mM DTT. After overnight dialysis to remove the excess dye, labeling efficiencies of >60% for each dye were calculated using the respective Cy3 and Cy5 extinction coefficients at 550 nm and 650 nm ( ϵ^Cy3^ 150 000 M^−1^ cm^−1^, ϵ^Cy5^ 250 000 M^−1^ cm^−1^). The labeled U2AF2 concentration was estimated from the calculated protein extinction coefficient (17 420 M^−1^ cm^−1^) following correction for the Cy3/Cy5 absorbances at 280 nm as we have described (39). The labeled U2AF2 was mixed with stoichiometric His_6_SumoMBP-U2AF1 and the salt concentration was reduced to 150 mM NaCl, 25 mM HEPES pH 7.0, 3% (v/v) glycerol, 0.2 mM TCEP by sequential dilutions with lower salt buffer. Lastly, the labeled complex was purified by Superdex-200 SEC in this buffer (Supplementary Figure S2). A mixture of Cy3/Cy5 fluorophores is expected at each U2AF2 site.

### Single molecule FRET data acquisition and analysis

Measurements were carried out at room temperature in 25 mM HEPES pH 7.0, 150 mM KCl, 0.01% (w/v) octaethylene glycol monododecyl ether, 0.2 mM TCEP, 6 mM β-mercaptoethanol, 1.5 mM Trolox (to eliminate Cy5 blinking), and an oxygen-scavenging system (0.8 mg/ml glucose oxidase, 0.625% (w/v) glucose, 0.02 mg/ml catalase). The sample chamber was assembled from quartz microscope slides and glass cover slips coated with a mixture of m-PEG and biotin–PEG and pre-treated with neutravidin (0.2 mg/ml). The U2AF2^Cy3/Cy5^ subunit concentration was similar in isolation and as in the heterodimer (5 nM). Excess U2AF1 (1000 nM) was mixed with the purified U2AF1–U2AF^Cy3/Cy5^ heterodimer (5 nM) before addition to the sample chamber and excess U2AF1 (1000 nM) also was included in the imaging buffer to ensure heterodimer formation. For surface tethering of the proteins *via* His_6_-tags on the U2AF2 subunit, the sample chamber was pre-incubated with 50 nM biotinylated (Ni^+2^)NTA resin (AnaSpec, Inc.). For surface tethering of the RNA sites, RNA splice sites were synthesized as 3′-conjugates with an 18-atom PEG spacer linked to a DNA oligonucleotide then pre-annealed with a complementary biotinyl-DNA oligonucleotide prior to incubation (at 10 nM for *AdML* or 50 nM for other splice sites) with the sample chamber. The sequence of the DNA oligonucleotide d(GTGCCAGCATATTTGTCGAAG) was carefully chosen from the *GAPA* gene of *E. coli* K12 to avoid potential homology to splice sites and lack energetically-favorable predicted secondary structure.

An Olympus IX71 inverted microscope, equipped with a UPlanApo 60×/1.20w objective lens was used for smFRET measurements. Cy3 and Cy5 fluorophores were excited with 532 and 642 nm lasers (Spectra-Physics). Total internal reflection (TIR) was obtained by a quartz prism (ESKMA Optics). The fluorescence emission was split into Cy3 and Cy5 fluorescence using a dual view imaging system DV2 (Photometrics) equipped with a 630 nm dichroic mirror and detected with an Andor iXon þ EMCCD camera. Movies were recorded using the Single software (courtesy of Prof. Taekjip Ha at John Hopkins U., http://ha.med.jhmi.edu/resources/) with an exposure time of 100 ms. We typically acquired up to five 5-min movies while imaging different sections of the slide for each sample. Before each measurement, we confirmed that non-specific binding was virtually absent by imaging U2AF2^Cy3/Cy5^ added to the slide in the absence of neutravidin.

Data sets were processed using scripts from http://ha.med.jhmi.edu/resources/ (Prof. T. Ha) with IDL and MATLAB software. Apparent FRET efficiencies (*E^app^*) were calculated using the equation *E^app^* = *I_Cy5_/(I_Cy5_+I_Cy3_)*; where *I_Cy3_* and *I_Cy5_* are the respective intensities of Cy3 and Cy5, using a MATLAB script generously provided by Prof. Peter Cornish (U. Missouri, Columbia). The FRET distribution histograms were built from traces that showed single photobleaching steps for Cy3 and Cy5 and/or anti-correlated events of donor and acceptor intensities. Anti-correlated changes in donor and acceptor intensities with constant sum of intensities indicated the presence of an energy transfer in single molecules labelled with one donor and one acceptor dye. All histograms were smoothed with a five- point window and plotted using Origin software (OriginLab Co.). At least three experimental replicates were collected for each sample. To facilitate comparison of different samples, the histograms were normalized such that the maximum count was set to one. The statistical significance of differences among the histograms were assessed for the two fitted peak areas (centered at 0.4 and 0.6), considering separate experiments as replicates, using Student's unpaired *t*-tests with Welch's correction as implemented in GraphPad Prism version 6.0 (Figures 6G and 7E).

### Fluorescence anisotropy equilibrium RNA affinity measurements

Apparent equilibrium dissociation constants (*K*_D_) of Supplementary Figures S2 and S5 were fit from changes in the fluorescence anisotropies of 5′-fluorescein (Fl)-labeled RNA oligonucleotides during titration with the indicated protein complexes as described (41). The binding buffer included 150 mM NaCl, 0.5 mM TCEP, 3% glycerol, 20 μM ZnCl_2_, 25 mM HEPES pH 6.8 and 0.2 U/μl SUPERase-In™ RNAse inhibitor. The Fl-*AdML* splice site sequence used for fluorescence anisotropy experiments is 5′-Fl-CUGUCCCUUUUUUUUCAC**AG** | CUCGCGGUUGAG, where ‘|’ marks the intron/exon junction) as given in Supplementary Figure S1A. The sequences of the Fl-*DEK(-3U)* and Fl-*DEK(-3C)* splice site RNA oligonucleotides used for the binding experiment in Supplementary Figure S5 were 5′-Fl-UAAGAAAUACUAAAUUAAUUUC**UAG**|AAAAGAGUCU and 5′-Fl-UAAGAAAUACUAAAUUAAUUUC**CAG**|AAAAGAGUCU, respectively. The Fl-*AdML* oligonucleotide is two nucleotides longer at the 5′ terminus and four nucleotides longer at the 3′ terminus than the *AdML* oligonucleotide used for smFRET. We consider the approximately five-fold higher affinity *K*_D_ (ΔΔ*G* ∼1 kcal mol^−1^) of the unlabeled MBP-tagged U2AF1–U2AF2 heterodimer measured by fluorescence anisotropy (120 ± 15 nM, Supplementary Figure S1) compared to the tethered MBP-tagged U2AF1–U2AF2^Cy3/Cy5^ (∼600 nM by comparison of 0.4 and 0.6 peak areas in the titration of Supplementary Figure S4) to be in reasonable agreement, considering avidity from binding six additional nucleotides of the longer Fl-*AdML* oligonucleotide.

## RESULTS

### U2AF1 stabilizes a high FRET conformation of U2AF2^Cy3/Cy5^

**Figure 2.**
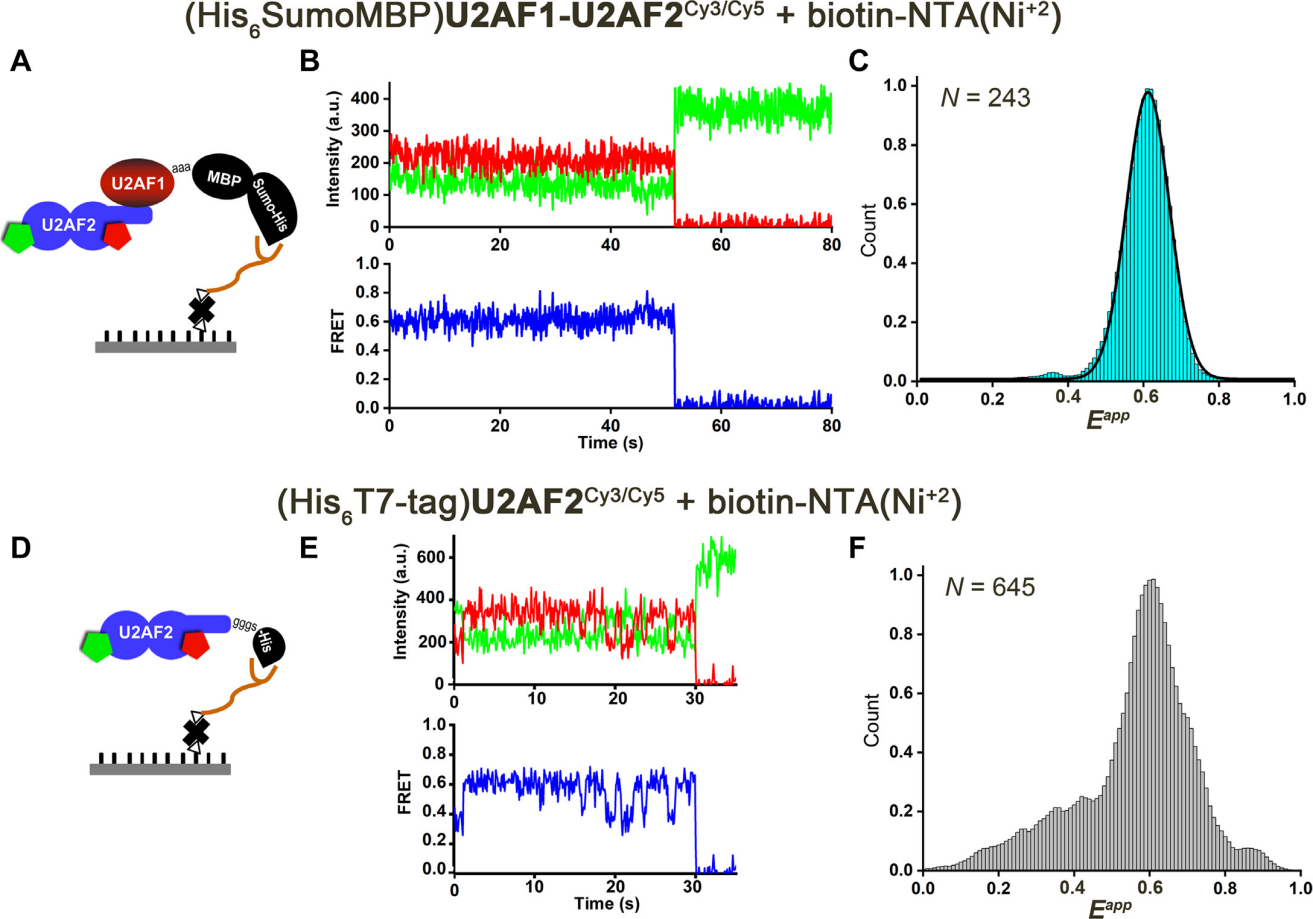
U2AF1 stabilizes a U2AF2 conformation with a high FRET value in the absence of RNA. (**A**) Scheme for U2AF1–U2AF2^Cy3/Cy5^ immobilization. The histidine-tagged U2AF1 (purified as a heterodimer with U2AF2^Cy3/Cy5^) was immobilized *via* biotinyl-NTA(Ni^+2^) on a neutravidin-coated surface. A mixture of Cy3/Cy5 is present at each labeled site. (**D**) The histidine-tagged U2AF2 subunit is immobilized directly as described (39). (**B**) Representative smFRET trace of the U2AF1–U2AF2^Cy3/Cy5^ heterodimer in absence of RNA show fluorescence intensities from Cy3 (green) and Cy5 (red) and the calculated apparent FRET efficiency (blue). The majority of U2AF1-bound U2AF2^Cy3/Cy5^ traces lack fluctuations and show predominate FRET values of ∼0.6. (**E**) Representative traces of the U2AF2 subunit alone, which is dynamic compared to the U2AF1-bound heterodimer. (C, F) Histograms compiled from *N* FRET traces showing the distribution of apparent FRET efficiencies (*E^app^*) for (**C**) U2AF1–U2AF2^Cy3/Cy5^ heterodimer (cyan) or (**F**) U2AF2^Cy3/Cy5^-only (gray). A black line represents the Gaussian fit of the U2AF1–U2AF2^Cy3/Cy5^ histogram in (E).

We leveraged our prior fluorophore sites in the U2AF2 RRMs (39) and methods to prepare nearly full length human U2AF1 (25,27) to investigate the influence of U2AF1 on the U2AF2 inter-RRM dynamics using smFRET. To study the U2AF heterodimer, we lengthened the U2AF2 construct to include the U2AF1 binding site (U2AF ligand motif, ULM) (Figure 1B). Unique cysteines introduced in the N-terminal RRM1 (A181C) and C-terminal RRM2 (Q324C) of U2AF2 were labeled with an equimolar mixture of Cy3 and Cy5 fluorophores as described (39). These fluorophore positions were chosen to maximize the expected differences in FRET efficiencies between the open (lower FRET) and closed (higher FRET) U2AF2 conformations (Figure 1D and E). The labeled U2AF2^Cy3/Cy5^ subunit was mixed with unlabeled, pre-purified U2AF1 subunit and the heterodimer was isolated following size exclusion chromatography (Supplementary Figure S2). An N-terminal His_6_SumoMBP tag tethered U2AF1 with bound U2AF2^Cy3/Cy5^ to a biotin-NTA/Ni^+2^ slide (Figures 1B and 2A). We compared the RNA binding affinities among various U2AF2 heterodimers with tagged or untagged U2AF1 constructs (diagrammed in Supplementary Figure S1A) by fitting changes in the fluorescence anisotropy of a fluorescein-tagged *AdML* RNA prototype (Figure 1F, Supplementary Figure S1B-F). The tag had no significant influence on the RNA binding affinity of the heterodimer. Moreover, inclusion of the zinc knuckle motifs enhanced the RNA binding affinity by 35-fold compared to the heterodimer of U2AF2 with the minimal U2AF1 UHM (ΔZn), which suggested that these regions are actively folded. The His_6_SumoMBP tagged U2AF1 construct (hereafter is referred to as U2AF1 for simplicity) reduced nonspecific interactions with the slide surface and was used for all FRET experiments. By tethering the labeled U2AF2^Cy3/Cy5^ subunit *via* U2AF1 (Figure 2A) and including excess unlabeled U2AF1 in the imaging buffer (Methods), we ensured that heterodimeric U2AF1–U2AF2^Cy3/Cy5^ complexes were imaged. For a well-controlled comparison with prior data, we also replicated smFRET experiments with the U2AF2^Cy3/Cy5^ subunit alone (Figure 2D) as described (39).

The presence of the U2AF1 subunit stabilized a high FRET state of U2AF2^Cy3/Cy5^. As previously observed using smFRET or small-angle X-ray scattering (39,42), the apo-U2AF2^Cy3/Cy5^ FRET distribution histogram was broad (Figure 2F). Consistent with our prior observations for this experimental system (39), approximately 30% of the apo-U2AF2^Cy3/Cy5^ smFRET traces showed transitions between different FRET values. The remaining 70% of the apo-U2AF2^Cy3/Cy5^ smFRET traces were static and mostly ∼0.6 FRET within the time resolution (0.1 sec) and typical time scale (0.1–50 s before Cy5 photobleaching) of our experiment (Figure 2E), which may omit extremely fast or slow transitions. By contrast, nearly all of the U2AF1–U2AF2^Cy3/Cy5^ traces (95%) showed stable FRET values of approximately 0.6 (Figure 2B). Accordingly, the FRET distribution histogram built from hundreds of U2AF1–U2AF2^Cy3/Cy5^ traces was narrowly centered at a FRET value of 0.6 (Figure 2C). We concluded that heterodimerization with the U2AF1 subunit stabilized a particular U2AF2 inter-RRM1/RRM2 arrangement with a characteristic high FRET value within the time scale that our smFRET experiment can resolve.

### A uridine-rich Py tract switches U2AF2^Cy3/Cy5^ to a U2AF1-stabilized lower FRET conformation

**Figure 3.**
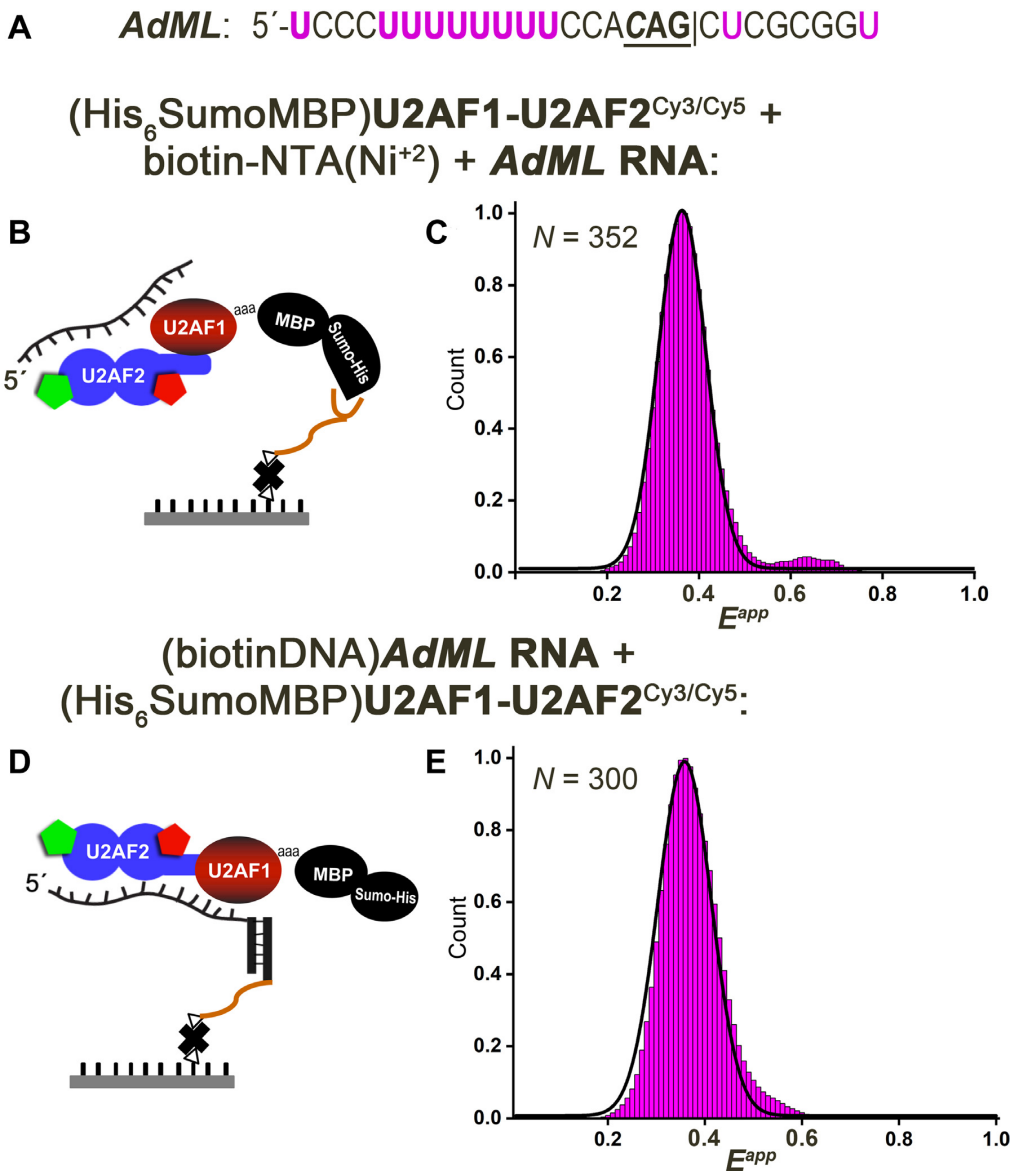
A uridine-rich, strong splice site RNA (*AdML*) switches the U2AF1–U2AF2^Cy3/Cy5^ heterodimer to a U2AF2 conformation corresponding to a lower FRET state. (**A**) Sequence of the *AdML* splice site oligonucleotide: ‘|’ spliced junction; three preceding nucleotides are bold and the -3 nucleotide is italicized. (**B**) Scheme for U2AF1–U2AF2^Cy3/Cy5^ protein immobilization and RNA addition, which is analogous to Figure 2A. (**C**) Histogram showing the distribution of apparent FRET efficiencies (*E^app^*) for tethered U2AF1–U2AF2^Cy3/Cy5^ following addition of *AdML* RNA. (**D**) Scheme for RNA immobilization and U2AF1–U2AF2^Cy3/Cy5^ protein addition. An RNA splice site-(PEG)-DNA conjugate was annealed with a biotin-DNA oligonucleotide and immobilized on a neutravidin-coated biotin-Peg surface. (**E**) Histogram showing the *E^app^* distribution for tethered *AdML* RNA bound to U2AF1–U2AF2^Cy3/Cy5^. Black lines represent the Gaussian fits of the histograms. *N*, total number of compiled single-molecule traces.

We then used smFRET to investigate the influence on U2AF2 conformations of binding to a strong, uridine-rich splice site from the adenovirus major late promoter prototype (*AdML*) (sequence given in Figure 3A). Remarkably, addition of the *AdML* RNA to the tethered U2AF1–U2AF2^Cy3/Cy5^ heterodimer switched the predominant 0.6 FRET state of the FRET distribution histogram to a narrow peak centered at an approximately 0.4 FRET value in a concentration-dependent manner (Figure 3B–C, Supplementary Figure S4). A similar 0.4 FRET state resulted from analysis of a reverse immobilization strategy, in which untethered U2AF1–U2AF2^Cy3/Cy5^ was added to AdML RNA attached to the slide (Figure 3D–E). The *AdML* RNA-bound heterodimer complex was extremely stable; indeed, 99% of the traces showed 0.4 FRET until photobleaching.

Addition of the isolated U2AF2^Cy3/Cy5^ subunit to the slide-tethered *AdML* splice site selectively increased a fraction of molecules with 0.4 FRET values (Supplementary Figure S3A–B), similar to our previous observations for this U2AF2 construct and the minimal *AdML* Py tract (39). Yet, the FRET distribution of the *AdML* RNA–bound U2AF2^Cy3/Cy5^ subunit remained broad and the traces often showed transitions (16% of traces), unlike the narrow FRET distribution and steady traces of the heterodimer. We concluded that a large, *AdML* RNA-dependent conformational change in U2AF2^Cy3/Cy5^ was stabilized by its heterodimer with U2AF1, independent of possible structural heterogeneity introduced by RNA-free proteins or tethering to the surface.

### A D215R/G319R mutant indicates that the high FRET state corresponds to closed U2AF2

**Figure 4.**
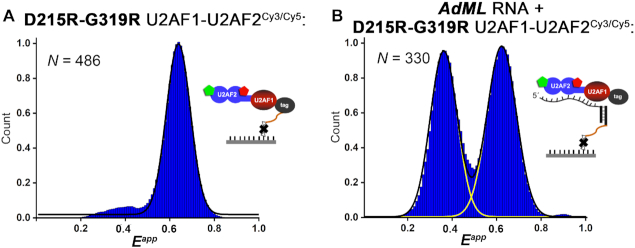
A D215R/G319R variant that is known to stabilize the closed U2AF2 conformation (40) promotes U2AF2^Cy3/Cy5^ conformations corresponding to the ∼0.6 FRET state. Histograms of the apparent FRET efficiencies (*E^app^*) for (**A**) tethered U2AF1–D215R/G319R U2AF2^Cy3/Cy5^ in the absence of RNA or (**B**) tethered *AdML* RNA in the presence of U2AF1–D215R/G319R U2AF2^Cy3/Cy5^. The immobilization strategies are inset. Black and yellow lines indicate the respective summed or individual Gaussian fits.

To investigate whether the 0.6 FRET state of U2AF2^Cy3/Cy5^ corresponds to the well-characterized closed conformation, we introduced a D215R/G319R double-mutation that strengthens the closed U2AF2 conformation by electrostatic complementarity (40) (Figure 1D–E). As expected, the already 0.6 FRET conformation of U2AF1–U2AF2^Cy3/Cy5^ appeared unchanged in the RNA-free histogram (Figure 4A). We then tested adding U2AF1–U2AF2^Cy3/Cy5^ to tethered *AdML* oligonucleotide, which ensured that only RNA-bound complexes were analyzed. Rather than shifting the entire U2AF2^Cy3/Cy5^ population to the lower 0.4 FRET value as observed for the wild-type heterodimer, approximately half of the conformational ensemble of the D215R/G319R mutant remained in the 0.6 FRET state (Figure 4B). This result supported our assignment of the 0.6 FRET state as the closed U2AF2 conformation, whereas the 0.4 FRET state is consistent with the open arrangement of U2AF RRM1/RRM2.

### Uridine-poor Py tracts bind open and closed U2AF1-stabilized U2AF2^Cy3/Cy5^ conformations

**Figure 5.**
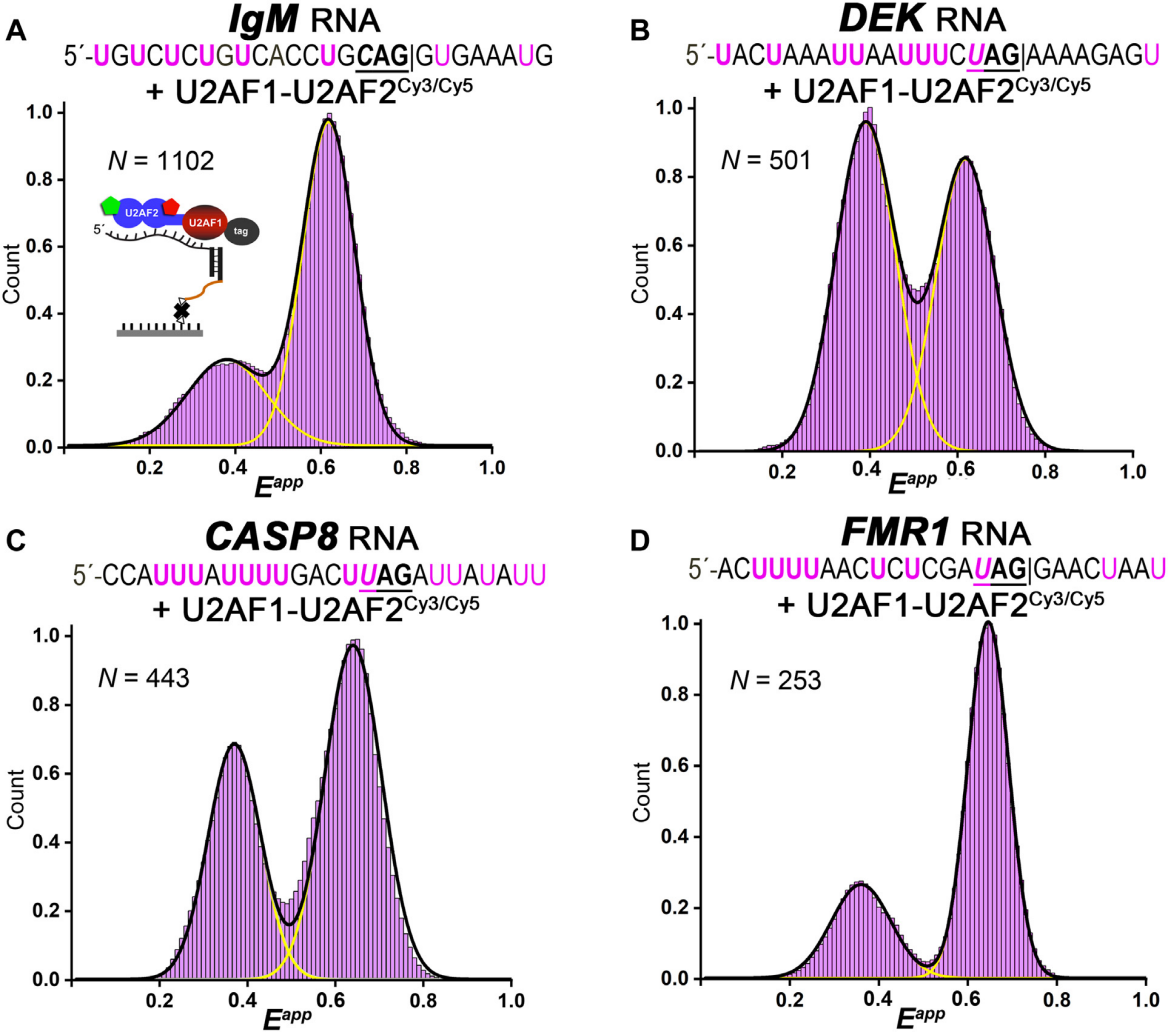
Binding to weak, uridine-poor splice sites alters the equilibrium between closed (0.6 FRET) and open (0.4 FRET) conformations of U2AF1–U2AF2^Cy3/Cy5^. Histograms showing the distribution of apparent FRET efficiencies (*E^app^*) for the respective (**A**) *IgM*, (**B**) *DEK* (also called *DEK(-3U)* in Figure 7), (**C**) *CASP8* or (**D**) *FMR1-*tethered complexes of U2AF1–U2AF2^Cy3/Cy5^ (lavender). The RNA is immobilized ensure that RNA-bound states are detected (scheme in panel A). The RNA sequences are given above each panel. Black and yellow lines indicate the respective summed or individual Gaussian fits.

Considering that the U2AF1 subunit is required for splicing a subset of weak splice sites with short, degenerate Py tracts, we next used smFRET to probe the influence of weak splice sites on the U2AF2 conformation in the U2AF1–U2AF2^Cy3/Cy5^ heterodimer (Figure 5). We started with a prototype of U2AF1-dependent splice sites from the immunoglobulin M gene (*IgM*) (46), and compared splice sites that have been shown to be affected by the MDS-associated S34F mutation (*DEK*, *CASP8*, and *FMR1*) (25,27). Apart from a previously documented stem-loop at the *IgM* 3′ splice site (47), no secondary structure was predicted for the other RNAs (Supplementary Table S1). The binding affinities of the heterodimer for these degenerate splice sites were very weak (e.g. *K*_D_ > 3 μM for *DEK* splice site in Supplementary Figure S5, which is 100-fold weaker than the ternary U2AF1–U2AF2–SF1 complex (25)). As such, we focused on the immobilization strategy of adding U2AF1–U2AF2^Cy3/Cy5^ heterodimer to the tethered RNAs (Figure 3D), which avoids introducing heterogeneity from RNA-free proteins. We also compared analogous smFRET experiments for the isolated U2AF2^Cy3/Cy5^ subunit bound to representative *IgM* and *DEK* RNAs and found broader but qualitatively similar histograms (Supplementary Figure S3C–E). Unlike the complete shift to a lower 0.4 FRET state observed for the heterodimer bound to strong *AdML* site, the *IgM* prototype of U2AF1-dependent splice sites binds most of the U2AF2^Cy3/Cy5^ molecules in the 0.6 FRET state corresponding to the closed conformation (∼70% of traces) (Figure 5A). The remaining population of traces (∼30%) shifts U2AF2^Cy3/Cy5^ to the 0.4 FRET state that are likely to correspond to the open conformation. Likewise, the smFRET distribution histograms built from hundreds of U2AF1–U2AF2^Cy3/Cy5^ traces bound to uridine-poor *DEK*, *CASP8* or *FMR1* splice sites showed populations of both 0.4 and 0.6 FRET traces at various ratios (Figure 5B–D). We concluded that populations of both open and closed U2AF2 conformations bind to splice sites with degenerate Py tracts.

Whereas apparent transitions between different FRET values were extremely rare for the U2AF1–U2AF2^Cy3/Cy5^ heterodimer bound to the uridine-rich *AdML* splice site, the U2AF1–U2AF2^Cy3/Cy5^ complexes with uridine-poor splice sites showed multiple spontaneous transitions between 0.4 and 0.6 FRET for 10–20% of traces. These characteristics of the heterodimer differed from the typically single, directional changes from ∼0.6 to ∼0.4 FRET that we had previously observed for the isolated U2AF2^Cy3/Cy5^ subunit binding to the *AdML* splice site (39). The single irreversible transitions from 0.6 to 0.4 FRET likely indicate that binding of the *AdML* splice site to closed U2AF2 induces a transition to the open U2AF2 conformation (39), which is compatible with this uridine-rich site. In contrast, the fluctuations of the U2AF1–U2AF2^Cy3/Cy5^ complexes with uridine-poor splice sites between 0.4 and 0.6 FRET may reflect the binding and unbinding of one RRM domain from these less compatible RNA sequences.

### The S34F mutation of U2AF1 modulates the U2AF2^Cy3/Cy5^ FRET distribution

**Figure 6.**
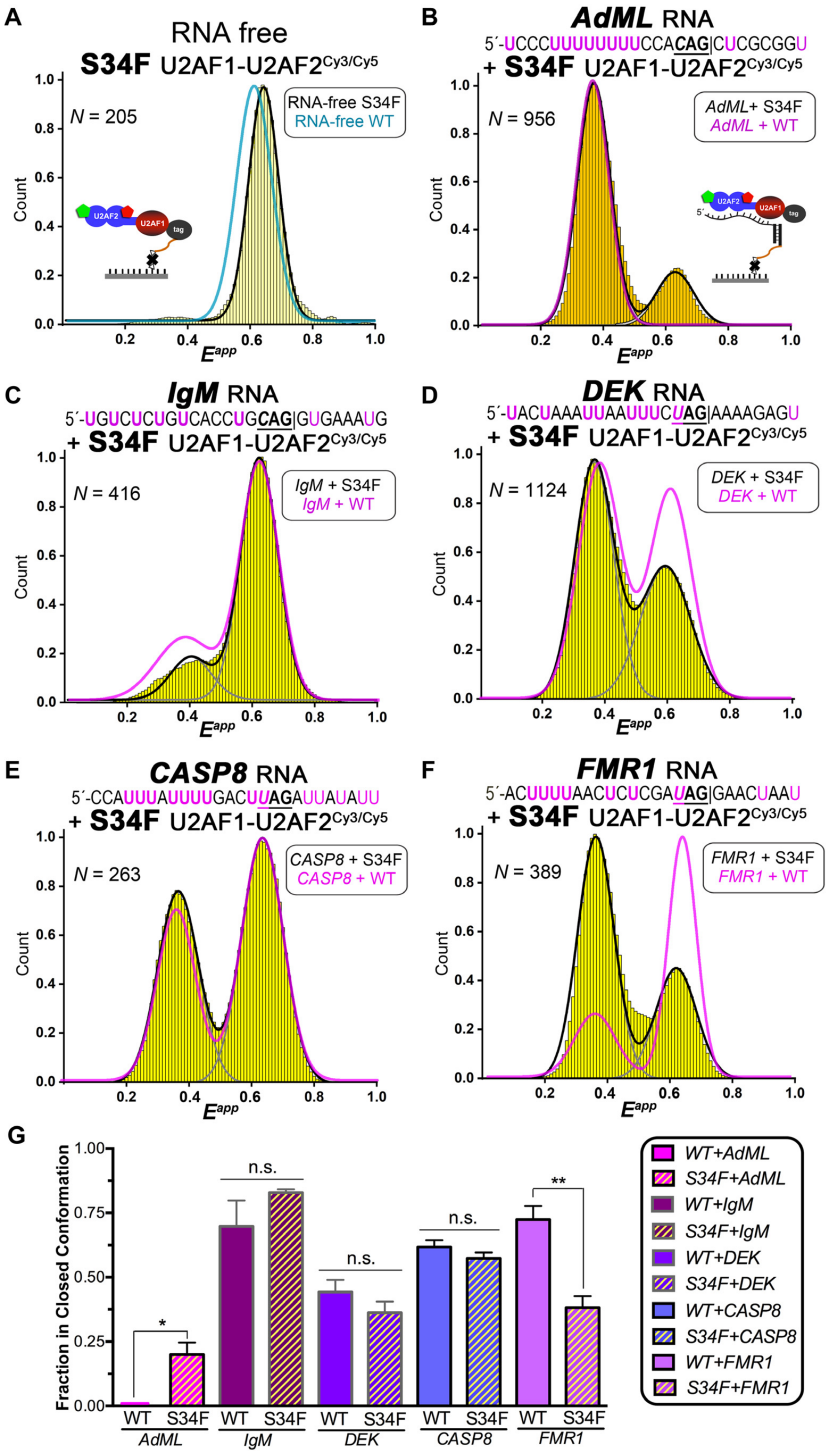
Influence of the MDS-associated S34F U2AF1 (yellow) on the U2AF1–U2AF2^Cy3/Cy5^ FRET distributions. Histograms of the apparent FRET efficiencies (*E^app^*) of the S34F-mutated U2AF1–U2AF2^Cy3/Cy5^ heterodimer either (**A**) in the absence of RNA, or (B–F) bound to tethered RNAs including (**B**) *AdML*, (**C**) *IgM*, (**D**) *DEK* (also called *DEK(-3U)* in Figure 7), (**E**) *CASP8*, (**F**) *FMR1*. The RNA-free protein in panel A is tethered by the His_6_-tag on U2AF1 as in Figure 2A (scheme inset). The RNA-bound proteins in panels B-F are tethered by annealing a DNA-linked splice site RNA with a biotin-DNA oligo as in Figure 3D (scheme inset in panel B). The splice site RNA sequences are given above the histograms. Black and gray lines indicate the respective summed or individual Gaussian fits of the S34F mutant histograms (widths < 0.14). The corresponding summed fits of wild-type U2AF1–U2AF2^Cy3/Cy5^ (pale cyan for RNA-free or magenta for RNA-bound) from Figures 2C, 3E and 6 are overlaid for comparison. (**G**) Bar graph showing the fraction of U2AF1–U2AF2^Cy3/Cy5^ in the closed conformation determined from the area under the Gaussian centered at 0.6 FRET normalized to the sum of Gaussians centered at 0.4 and 0.6 FRET. The average values and standard deviations among three experimental replicates are plotted. The results of two-tailed unpaired *t*-tests with Welch's correction are indicated: Not significant (n.s.) *P*> 0.05; *P< 0.05; **P< 0.005.

The MDS-associated S34F mutation occurs at a putative U2AF1–RNA interface and could influence the conformation of the U2AF2 subunit for Py tract recognition. We used smFRET to investigate the effects of the U2AF1 S34F mutation on the U2AF2^Cy3/Cy5^ conformations, first in the absence of RNA and then bound to the uridine-rich *AdML* or various uridine-poor splice sites (Figure 6). The S34F mutation appeared to stabilize the RNA-free S34F mutant heterodimer in the apparent 0.6 FRET state (Figure 6A) and significantly increased the population of U2AF1–U2AF2^Cy3/Cy5^ complexes bound to the AdML splice site in this state (from essentially none to 25% of the observed values, Figure 6B and G). Yet, no consistent S34F-associated trend is observed among U2AF1–U2AF2^Cy3/Cy5^ complexes with different splice sites. The FRET distributions of the S34F mutant compared to wild-type U2AF1–U2AF2^Cy3/Cy5^ complexes with *IgM*, *DEK* or *CASP8* splice site RNAs remained similar (Figure 6C–E). Moreover, the S34F U2AF1 mutant significantly promoted the open (rather than closed) conformation of the *FMR1* splice site complex (66% closed for the wild-type *FMR1* complex compared to 33% for the S34F mutant, Figure 6F and G).

### U2AF1-stabilized U2AF2^Cy3/Cy5^ conformations sense the -3C/U preceding the spliced junction

**Figure 7.**
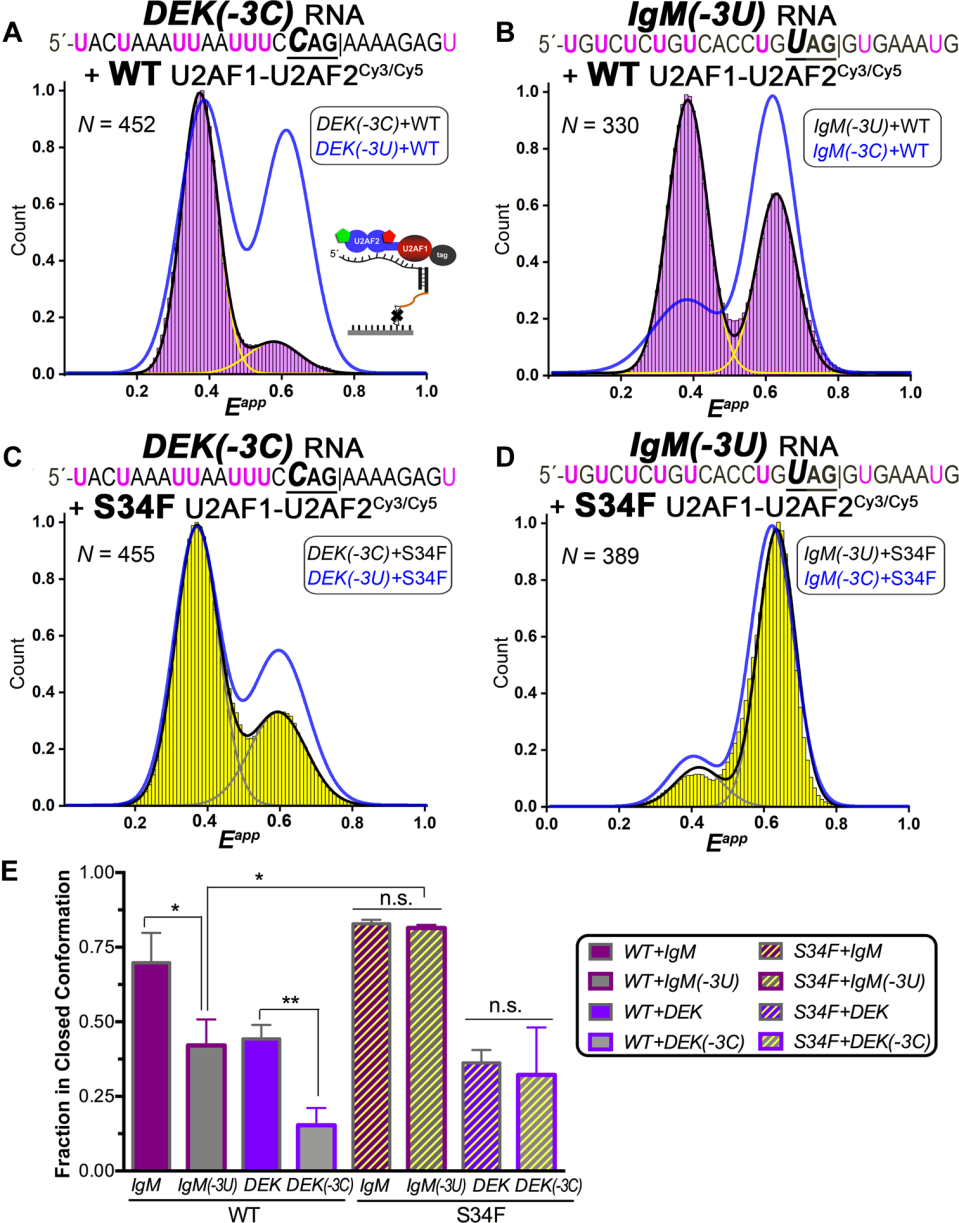
Influence of a -3C *versus* -3U nucleotide preceding the splice site junction on the of apparent FRET efficiencies (*E^app^*) of the wild-type (WT) and S34F mutant U2AF1–U2AF2^Cy3/Cy5^ heterodimers, including (**A**) WT U2AF1–U2AF2^Cy3/Cy5^ bound to *DEK(-3C)* RNA, (**B**) WT U2AF1–U2AF2^Cy3/Cy5^ bound to *IgM(-3U)* RNA, (**C**) S34F U2AF1–U2AF2^Cy3/Cy5^ bound to *DEK(-3C)* RNA, (**D**) S34F U2AF1–U2AF2^Cy3/Cy5^ bound to *IgM(-3U)* RNA. The ribonucleoproteins are tethered *via* the bound RNAs as inset in panel A and shown in Figure 3D. Black lines indicate the summed Gaussian fits of the histograms, and yellow or gray lines indicate the individual fits. The summed fits of the FRET histograms for the natural splice sites (*DEK(-3U)* or *IgM(-3C)*) bound to the corresponding WT or S34F mutant heterodimers are overlaid in blue for comparison. (**E**) Bar graph showing the fraction of U2AF1–U2AF2^Cy3/Cy5^ in the closed conformation determined from the area under the Gaussian centered at 0.6 FRET normalized to the sum of Gaussians centered at 0.4 and 0.6 FRET, plotted as in Figure 6G.

As shown by RNAseq and RNA binding experiments (25–27) (Figure 1F, Supplementary Figure S1F), human U2AF1 slightly prefers a cytidine compared to a uridine located three nucleotides preceding that splice site junction and this preference is enhanced by the S34F mutation. Since the arrangement of the splice site signals relative to the U2AF1 and U2AF2 subunits are unknown, it is possible that U2AF2 contacts the –3C/U nucleotide and is influenced indirectly by the U2AF1 subunit and its S34F mutation. We first investigated the influence of a -3C variant for the *DEK* splice site, which normally is preceded by a –3U, on the conformational ensemble of the isolated U2AF2^Cy3/Cy5^ subunit. The *DEK(-3C)* variant preferentially associated with the open U2AF2^Cy3/Cy5^ conformation, which differed from the open and closed U2AF2^Cy3/Cy5^ populations bound to the original *DEK(-3U)* RNA (Supplementary Figure S3D–E). As for other splice sites, inclusion of the U2AF1 subunit sharpened the FRET distributions of U2AF2^Cy3/Cy5^ but did not change the relative preference for 0.6 compared to 0.4 FRET values (Figure 7). Binding to the *DEK(-3C)* RNA increased the population of open U2AF1–U2AF2^Cy3/Cy5^ conformations relative to the original *DEK(-3U)* splice site (Figure 7A). We next extended our experiments to a –3U-substituted *IgM* splice site, which naturally is preceded by a –3C. The artificial *IgM(-3U)* nucleotide again increased the population of open U2AF1–U2AF2^Cy3/Cy5^ conformations compared to the natural *IgM(–3C)* parent sequence (Figure 7B), despite a C-to-U change for the artificial *IgM* rather than the opposite U-to-C variation of the *DEK* splice site.

We then compared the influence of the S34F mutation on the FRET distributions of the U2AF1–U2AF2^Cy3/Cy5^ heterodimer bound to the –3U/C variant splice sites. The differences between the *DEK(–3C)* compared to the *DEK(–3U)* splice site complexes were more subtle for the S34F mutant compared to the wild-type heterodimer (Figure 7C), although the open, ∼0.4 FRET population still increased slightly for the S34F-mutant U2AF1–U2AF2^Cy3/Cy5^ bound to artificial *DEK(–3C)* RNA compared to the original splice site. For the S34F mutant heterodimer bound to *IgM(–3U)* RNA, the FRET distribution histograms remained indistinguishable compared to the *IgM(–3C)* splice site (Figure 7D). We concluded that in general, the open/closed U2AF2 conformations in the wild-type U2AF1 heterodimer normally sense the cytidine/uridine identity at the –3 position of the splice sites and are influenced by the RNA sequence of the surrounding splice site. Remarkably, the S34F mutation reduced the ability of U2AF2 to conformationally respond to the -3U/C identities of the splice sites tested here.

## DISCUSSION

**Figure 8.**
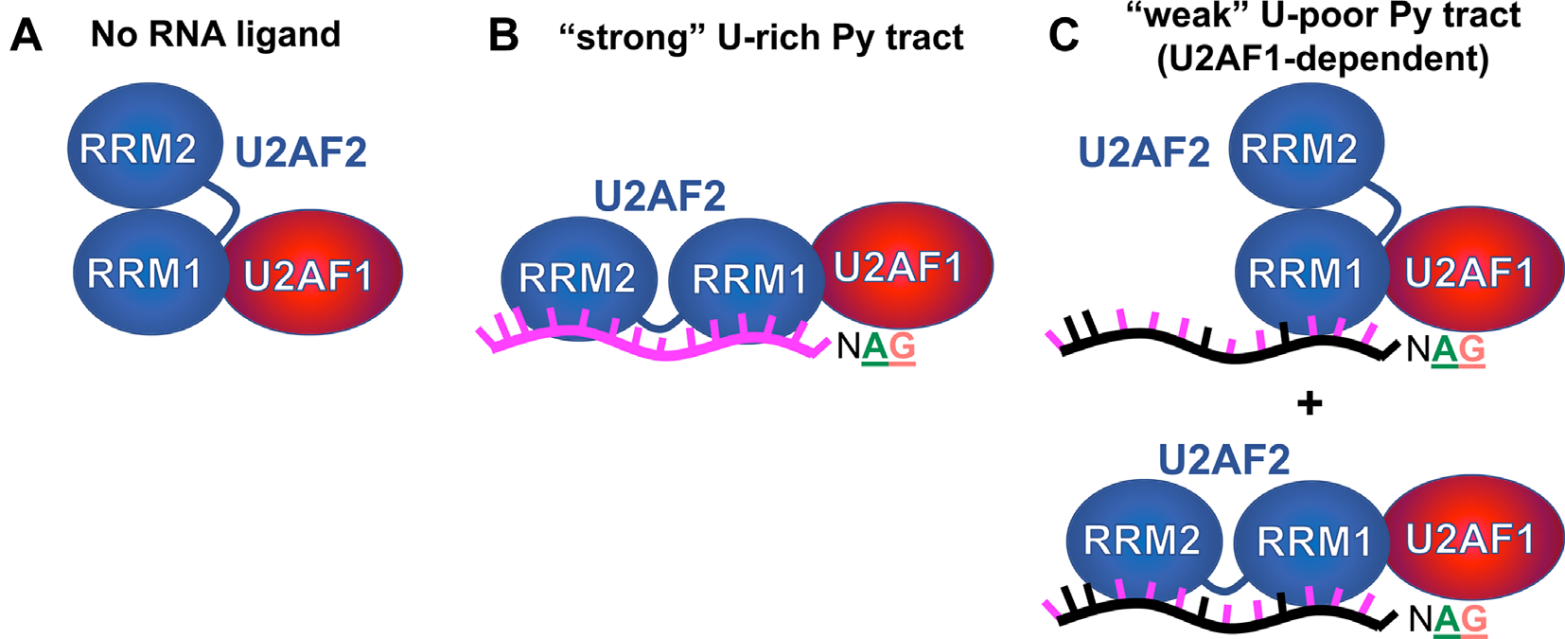
Models for splice site RNAs switching of the conformations of U2AF2 in the U2AF1–U2AF2 heterodimer. (**A**) The closed U2AF2 conformation predominates in the absence of RNA ligand. (**B**) Binding to a U-rich splice site RNA switches U2AF2 to the open, side-by-side RRM configurations. (**C**) A degenerate splice site signal with a short, interrupted Py tract preceding the AG-dinucleotide binds populations of closed and open conformations. The arrangements of RRM1/RRM2 in the schematic diagrams are based on PDB ID’s 2YH0 (closed) and 2YH1/5EV4 (open).

Our smFRET data reveals RNA-responsive switching between the open and closed conformations of human U2AF2 that is stabilized in the context of U2AF1–U2AF2 heterodimer (Figure 8). Consistent with prior results (as further discussed below), the isolated U2AF2^Cy3/Cy5^ subunit is conformationally dynamic and shows a continuum of less populated inter-RRM1/RRM2 distances in the smFRET histograms ((39,44), this work). Heterodimerization with the U2AF1 subunit reinforces distinct structural states of U2AF2. The narrow FRET distributions of the U2AF1–U2AF2^Cy3/Cy5^ heterodimer show two major close-packed arrangements of U2AF2 RRM1/RRM2 corresponding to the closed and open conformations, although extended conformations in which the two U2AF2 RRMs fully dissociate may exist beyond the detectable limit of our smFRET experiments. The U2AF2 conformations of the U2AF heterodimer and its ribonucleoprotein complexes are remarkably stable and rarely show dynamic transitions in the smFRET traces in the time frame of our smFRET experiments (∼0.1 to 50 s before photobleaching). Notably, this >50 sec estimate for the U2AF2 conformational lifetime in the heterodimer would be sufficient to initiate splicing, considering that most mammalian introns are spliced in ∼0.5–3 min (reviewed in (48)). As such, the smFRET results for the U2AF1–U2AF2^Cy3/Cy5^ heterodimer suggest a new structural role for the U2AF1 subunit to stabilize the U2AF2 conformation over a sufficient time frame to promote spliceosome assembly. Although we do not detect an S34F-associated change in U2AF2 dynamics within experimental constraints, it remains possible that stabilization of U2AF2 conformations could contribute to a kinetic influence of the U2AF1 protein and its S34F mutation on splicing and release of nascent transcripts from the RNA polymerase complex (49).

Both the open and closed conformations of U2AF2 in the U2AF heterodimer associate with natural, U2AF1-sensitive splice site RNAs. Since nearly all of the RNA oligonucleotides studied here are predicted to be single-stranded (except one well-characterized stem-loop of *IgM*, Supplementary Table S1), the splice site-sensitive differences in U2AF2 conformations are unlikely to depend on RNA structures. The populations of the open and closed conformations also lack obvious correlation with the *total* number of uridines in the RNA ligands. Hints emerge when considering the number of uridines in the putative binding site of the U2AF2 RRMs, which is likely to be located –5 to –13 nucleotides preceding the splice junction. For example, the closed U2AF2 conformation dominates when the heterodimer is bound to the *IgM* and *FMR1* splice sites (three uridines within –5 to –13 nucleotides), whereas the population of open U2AF2 conformations increases in complexes of the heterodimer with the *DEK* or *CASP8* (five uridines within –5 to –13 nucleotides). Nevertheless, predictive rules are challenging to derive in the absence of high resolution structural information on the relative binding sites of the U2AF domains and the 3′ splice site.

The results reported here agree with prior structural and biophysical studies of the U2AF2 RRM1/RRM2 containing region, despite differences among the construct boundaries and complementary strengths of diverse techniques. In the crystal structure of the open U2AF2 conformation, N-/C-terminal extensions of the RRM1/RRM2 domains form α-helices that contact the RNA and the inter-RRM linker (39). These extensions were truncated in our earliest SAXS characterization of the U2AF2 RRM1/RRM2 region (41), and the N-terminal extension continued to be absent from most subsequent SAXS, NMR or FRET experiments (37,40,43,44). Yet even including the RRM1/RRM2 extensions, the isolated U2AF2 subunit remains conformationally heterogeneous ((39,42,44), this work). As confirmed here, binding to a strong, uridine-tract increases the population of U2AF2 molecules with FRET values corresponding to the open RRM1/RRM2 conformation (39,44). Adenosine- or cytidine-interrupted uridine tracts have been previously observed to shift the populations of U2AF2 conformations (37,44), hinting at the sensitivity of the U2AF2 conformations to different splice site sequences here noted and shown to be reinforced by the heterodimer with U2AF1.

Our comparison with the D215R/G319R U2AF2^Cy3/Cy5^ mutant clarifies that the U2AF1-stabilized 0.6 FRET state of U2AF2^Cy3/Cy5^ corresponds on average to closed RNA-free U2AF2, whereas the 0.4 FRET state is consistent with open U2AF2 bound to a strong uridine-tract, and populations of both open and closed conformations bind to natural, uridine-poor splice sites. This does not rule out the possibility of dynamic conformational switching on a submillisecond time scale, such as observed for the U2AF2 subunit (44). The FRET populations of the U2AF2^Cy3/Cy5^ heterodimer with U2AF1 differ from those reported for the heterodimer with the minimal U2AF1 UHM, which was found to shift the RNA-free or RNA-bound conformational ensemble of the U2AF2 RRM1/RRM2 towards the open conformation (44). As such, the U2AF1 zinc knuckles influence the U2AF2 inter-RRM conformation either directly or allosterically. In support of a direct interface between U2AF1 and the U2AF2 RRM1, the U2AF1 UHM shows strong intermolecular PRE’s and tumbles as a unit with the U2AF2 RRM1 (44). The U2AF1 zinc knuckles fold tightly on the UHM surface (33) (Figure 1C) and may be located close to the U2AF2 RRM1, although this idea awaits structural proof.

The presence of the U2AF1 zinc knuckles in the U2AF1–U2AF2^Cy3/Cy5^ heterodimer enabled us to probe the influence of the MDS-associated S34F mutation of U2AF1. This common U2AF1 mutation is distinct from the rare, MDS-associated U2AF2 L187V and M144I mutations, which lacked apparent differences on the NMR spectra or RNA binding of U2AF2 (44). Out of the seven splice site complexes examined here, the S34F mutation induced significant shifts in the populations of U2AF2 conformations bound to *FMR1*, *AdML* or *IgM(–3U)* RNAs. The S34F mutation favored the open U2AF2 conformation when bound to the *FMR1* site but closed for the *IgM(–3U)* or *AdML* complexes. The reason for these RNA sequence-dependent differences in the effects of the S34F mutation are not certain. One conjecture is that the bulky phenylalanine might contribute favorable hydrophobic contacts to the closed U2AF2 conformation bound to an optimal splice site such as *AdML*, yet strain closed U2AF2 in the context of other splice site RNA complexes. For example, if the phenylalanine side chain strains the closed U2AF2 conformation, the *FMR1* site offers an upstream uridine tract for docking of the open U2AF2 RRM2 whereas *IgM* lacks a uridine tract. Since the S34F mutation appears to block –3U/C sensing by the U2AF2 conformations, it is less surprising that U2AF2 in the S34F mutant heterodimer primarily remains closed when bound to either *IgM* and *IgM(–3U)*, whereas the open U2AF2 population of the wild-type heterodimer increases following introduction of the –3U. We conclude that the S34F mutation can, but does not as a rule, influence the population of U2AF2 conformations.

A major outstanding question is whether one or both U2AF2 conformations are functionally active in gene expression pathways supported by the heterodimer. For spliceosome assembly, one possibility is that only the open conformation of U2AF2 supports annealing of the U2 snRNA with the branch site (50), whereas the closed conformation could block splicing. Since the populations of each U2AF2 conformation depend on the degeneracy of the Py tract sequence, Py tract-sensing conformations with different activities for spliceosome recruitment could provide a checkpoint for splice site fidelity and also explain the different splicing efficiencies of uridine-rich and uridine-poor splice sites. The open U2AF2 population and hence splicing activity would be expected to increase following association of the U2AF heterodimer with uridine-rich Py tract signals such as *AdML*. Conversely if the closed fraction of U2AF2 molecules is inactive, a mixture of closed and open U2AF2 conformations would decrease the net splicing activity of uridine-poor Py tract signals. Although the U2AF2 conformations themselves do not appear tunable in response to RNA sequence (only the populations of two conformational classes are modified), different ratios of active/inactive structures would enable U2AF2 to act as a graded rheostat for tunable splicing in response to different splice site signals. In support of this idea, the D215R/G319R mutant U2AF2 lacks detectable activity for spliceosome assembly in biochemical reconstitution assays with the highly efficient *AdML* substrate (44). Nevertheless, it remains possible that the D215R/G319R mutant directly rather than conformationally interferes with requisite interactions for the complicated spliceosome assembly process, and/or that closed U2AF2 functions in the context of splice site sequences with weaker Py tract signals.

With these considerations, it remains possible that both open and closed U2AF2 conformations can initiate spliceosome assembly. Based on our detection of only two well-defined U2AF2 states among the RNA-free heterodimer and seven splice site complexes, our prior proposal that these conformations serve to adapt the U2AF2 structure to fit the broad range of human splice site signals appears unlikely (37). Yet, an intriguing possibility remains that the U2AF1-dependent class of uridine-poor splice sites (also called AG-dependent) may be spliced primarily through the closed U2AF2 conformation, whereas uridine-rich U2AF1-independent sites would be spliced *via* open U2AF2. In a minor U12-type spliceosome that entirely lacks Py tract signals, the U2AF1-like ZRSR2 subunit contains a heterodimerization domain that is expected to bind a U2AF2-like paralogue (15). One may suppose that a yet-to-be-identified U2AF2 subunit of the minor spliceosome would recognize the uridine-poor U12-type splice sites primarily in the closed conformation. Moreover, we suggest that the ratios of open/closed conformations observed here for human U2AF2 could differ from homologues that bind different consensus Py tracts. For example, we anticipate that the closed U2AF2 conformation might dominate recognition of the negligible or very short U-tracts of *Neurospora crassa* and *Caenorhabditis elegans* splice sites, which also are highly dependent on the U2AF1 subunit for binding and splicing (4,51,52).

Regulation of non-splicing functions by RNA sequences modulating ratios open/closed U2AF2 conformations also remains an open question. Translational repression is mediated in part by binding of the U2AF heterodimer or its U2AF26 paralogue to splice site-like mRNA sequences (7,8), which based on the results here, would be expected to influence the ratio of open/closed U2AF2 conformations. Likewise, alternative polyadenylation, involves U2AF2/U2AF1 interactions not only with cleavage factors (28,53) but also in certain cases, with RNA sequence elements in noncanonical 3′ end signals (9,11). Moreover, both translation and polyadenylation has been shown to be altered by the S34F mutation (7,28), which could act in part by modulating the ratios of U2AF2 conformations bound to the RNA sites of these pathways, as observed for the *FMR1*, *AdML* and *IgM(-3U)* splice sites here.

In cells, a third splicing factor subunit called SF1 binds to U2AF2 as a ternary complex with U2AF1 and recognizes the branch site splicing signal upstream of the Py tract. Here, we focused on the minimal U2AF2 regions for U2AF1 heterodimerization and Py tract recognition, which avoids the potential complications of labeling full length U2AF2 in the presence of its the cysteine-rich SF1-interaction domain. The field will benefit from future studies of the conformational dynamics among the participating subunits of the intact U2AF1–U2AF2–SF1–splice site RNA complex, as well as distinguishing the functional activities of open/closed U2AF2, exploring the conformational evolution of U2AF2 homologues and potential paralogues in the U12-type spliceosome, and high resolution structures of these important 3′ splice site complexes.

## Supplementary Material

gkaa293_Supplemental_File
