# Supplementary material for: A splice site-sensing conformational switch in U2AF2 is modulated by U2AF1 and its recurrent myelodysplasia-associated mutation
Source: Nucleic Acids Res. 2020 Apr 28;48(10):5695–709. doi: 10.1093/nar/gkaa293 (PMC7261175; doi:10.1093/nar/gkaa293)
Supplement: gkaa293_Supplemental_File [file gkaa293_supplemental_file.pdf]

Supplementary Table S1. Stabilities of most favorable predicted RNA secondary structures.

Splice site:	RNA sequence*:	RNAstructure [1]
AdML	UCCCUUUUUUUUCCA**<u>C</u>AG** | CUCGCGGU	>0
IgM	UGUCUCUGUCACCUG**<u>C</u>AG** | GUGAAAUG	-5.6 kcal mol^{-1}
IgM(-3U)	UGUCUCUGUCACCUG**<u>U</u>AG** | GUGAAAUG	-5.7 kcal mol^{-1}
DEK	UACUAAAUUAAUUUC**<u>U</u>AG** | AAAAGAGU	>0
DEK(-3C)	UACUAAAUUAAUUUC**<u>C</u>AG** | AAAAGAGU	>0
FMR1	ACUUUUAACUCUCGA**<u>U</u>AG** | GAACUAAU	>0
CASP8	CCAUUUAUUUUGACU**<u>U</u>AG** | AUUAUAUU	-0.1 kcal mol^{-1}
Biotinyl-DNA	d(GTGCCAGCATATTTGTCGAAG)	>0

*Bold font marks the consensus AG dinucleotide; "|" marks intron-exon junction; Bold and underlined font marks -3 nucleotide relative to the exon. Predicted energies of ">0" indicate that base pairing is less favored than random coil. The biotinyl-DNA is conjugated via an 18-atom polyethylene glycol linker to the RNA splice site.

1. Reuter, J.S. and Mathews, D.H. (2010) RNAstructure: software for RNA secondary structure prediction and analysis. *BMC Bioinformatics*, **11**, 129.

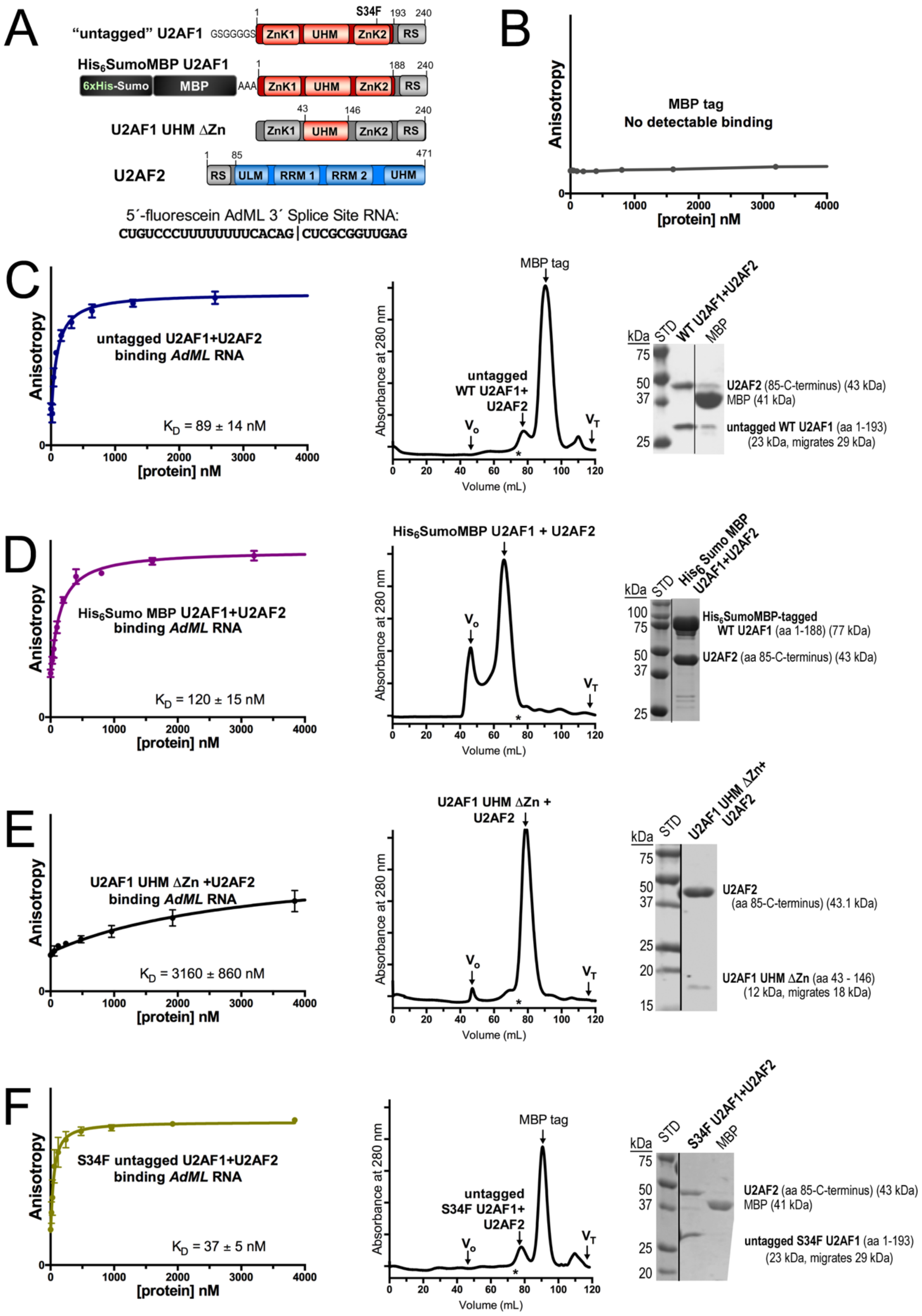

Supplementary Figure S1 legend follows on next page.

Supplementary Figure S1 ***(preceding page).*** Fluorescence anisotropy curves and protein preparations corresponding to Figure 1F. (**A**) Schematic diagram of protein constructs. Colored regions were included. The sequence of the fluorescein (Fl)-labeled *AdML* RNA oligonucleotide used in the fluorescence anisotropy binding experiments is given below. As described in the methods, all U2AF2 subunits included amino acids (aa) 85-371 (C-terminus) of NCBI RefSeq NP_001012496. The untagged wild-type (WT) or S34F U2AF1 constructs (aa 1-193) were expressed as N-terminal fusion with an MBP tag followed by a TEV protease site and GSGGGGS linker that remains following cleavage to release the tag. The His_6SumoMBP-tagged U2AF1 was linked by a three alanines and included aa 1-188 of U2AF1 Refseq NP_006749, for which a serine was substituted for a poorly conserved cysteine (C67S) and a C-terminal alanine was added to enhance soluble protein expression. The U2AF1 UHM (ΔZn, aa 43 to 146 with C67S) was purified separately from U2AF2, mixed and purified by size exclusion chromatography (SEC). (**B**) The MBP tag itself (prepared as in C) does not detectably bind Fl-*AdML* RNA. (**C** - **F**) *Left:* Fluorescence anisotropy changes from titration of the indicated protein complex into Fl-*AdML* RNA. Inset are apparent equilibrium dissociation constants (K_D) and standard deviations of three replicated *AdML* RNA binding experiments for each wild-type U2AF heterodimer variant and six for the S34F mutant. *Middle:* SEC chromatogram of the indicated heterodimer on a HiLoad 16/60 Superdex 200 pg column (GE Healthcare, Inc.). V_o, void volume where aggregates would be expected; V_T, total column volume. An asterisk marks the expected elution volume of conalbumin, a molecular size standard. The peak identities are labeled. *Right:* SDS-PAGE of pooled peak fractions compared with size standards including relevant preparative steps. Gels are Coomassie-blue stained although scanned in grayscale. Samples include (**C**) untagged WT or (**D**) S34F heterodimer, (**E**) His_6SumoMBP-tagged U2AF1+U2AF2 heterodimer, or (**F**) untagged U2AF1 UHM ΔZn (without zinc knuckles)+U2AF2.

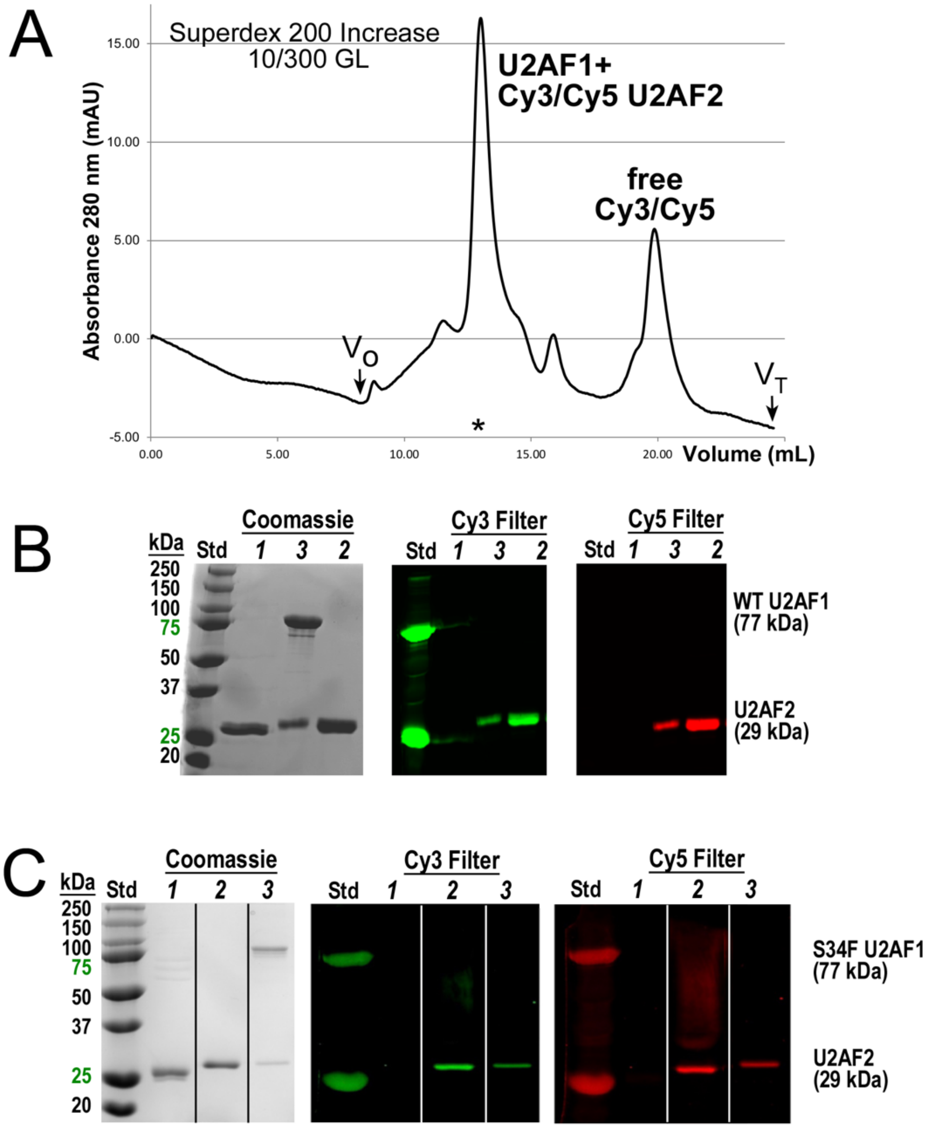

Supplementary Figure S2. Final preparation of wild-type (WT) or S34F mutant His$_6$SumoMBP-U2AF1–U2AF2$^{Cy3/Cy5}$ heterodimer for smFRET. The protein constructs are diagrammed in Figure 1B. **(A)** Chromatogram of WT His$_6$SumoMBP-U2AF1 mixed with U2AF2$^{Cy3/Cy5}$ and separated from unreacted dye using a Superdex-200 Increase 10/300 GL column (GE Healthcare, Inc.). V$_o$, void volume; V$_T$, total column volume. An asterisk (*) marks the expected elution volume of an aldolase size standard (158 kDa) and is consistent with the elution peak of the His$_6$SumoMBP-U2AF1–U2AF2$^{Cy3/Cy5}$ heterodimer (106 kDa), considering different hydrodynamic radii and column resolution. The elution volume of the S34F U2AF1–U2AF2$^{Cy3/Cy5}$ heterodimer is indistinguishable from WT. **(B-C)** SDS-PAGE of WT (**B**) or S34F mutant (**C**) preparations for smFRET, imaged with Coomassie blue stain scanned in grayscale (*Left*), Cy3 filter (*Middle*), or Cy5 filter (*Right*). Lanes correspond to: *1* unlabeled U2AF2; *2*, labeled U2AF2$^{Cy3/Cy5}$ following dialysis, *2,* final His$_6$SumoMBP-U2AF1–U2AF2$^{Cy3/Cy5}$ heterodimer after size exclusion chromatography. A mixture of Cy3/Cy5 is expected at each U2AF2 site.

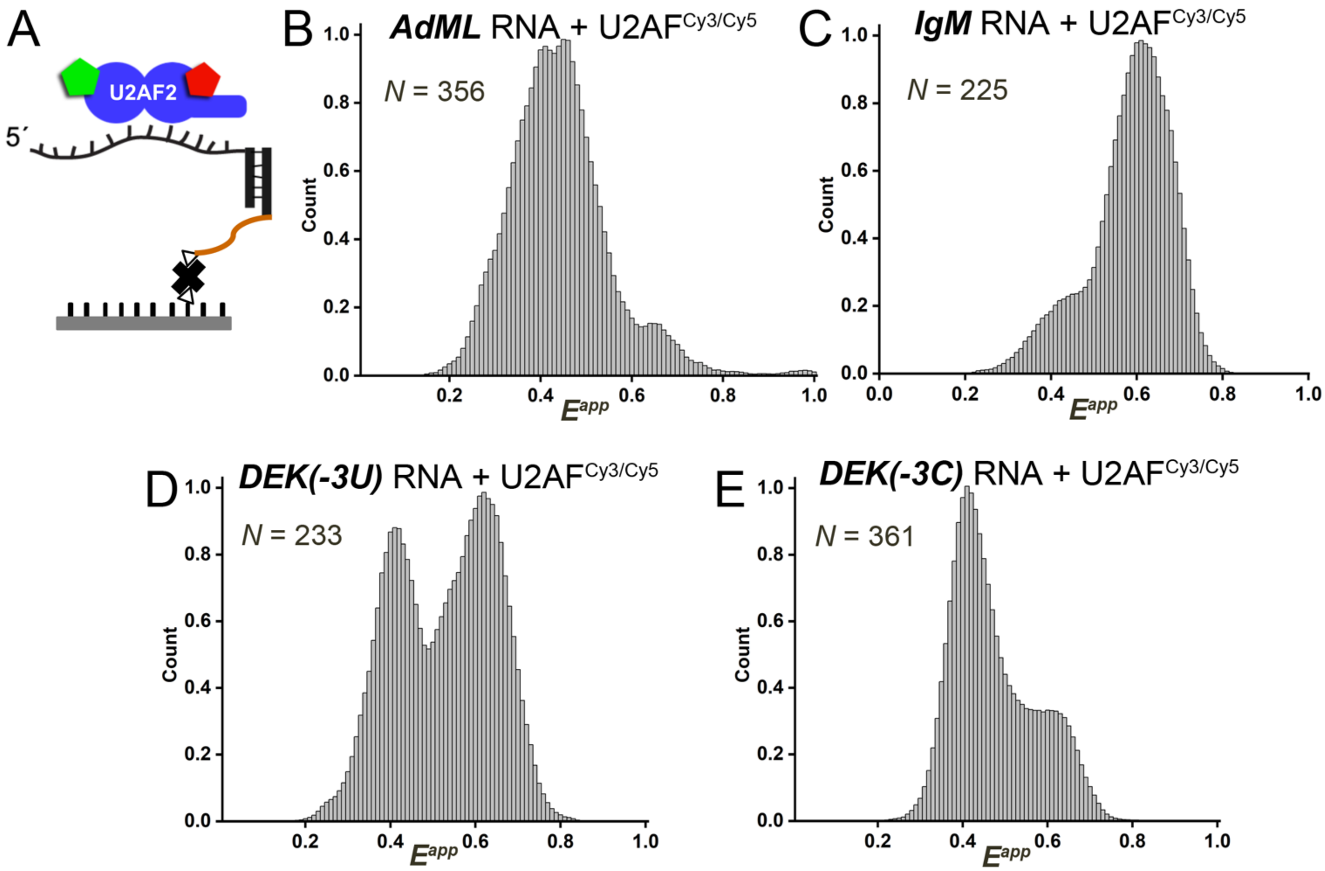

Supplementary Figure S3. Apparent FRET of RNA complexes with the isolated U2AF2$^{Cy3/Cy5}$ subunit. (**A**) Scheme for splice site RNA immobilization and U2AF2$^{Cy3/Cy5}$ protein addition. (**B - E**) Histograms showing the distribution of apparent FRET efficiencies (E_{app}) for the untethered U2AF2$^{Cy3/Cy5}$ subunit bound to the indicated, slide-tethered RNA.

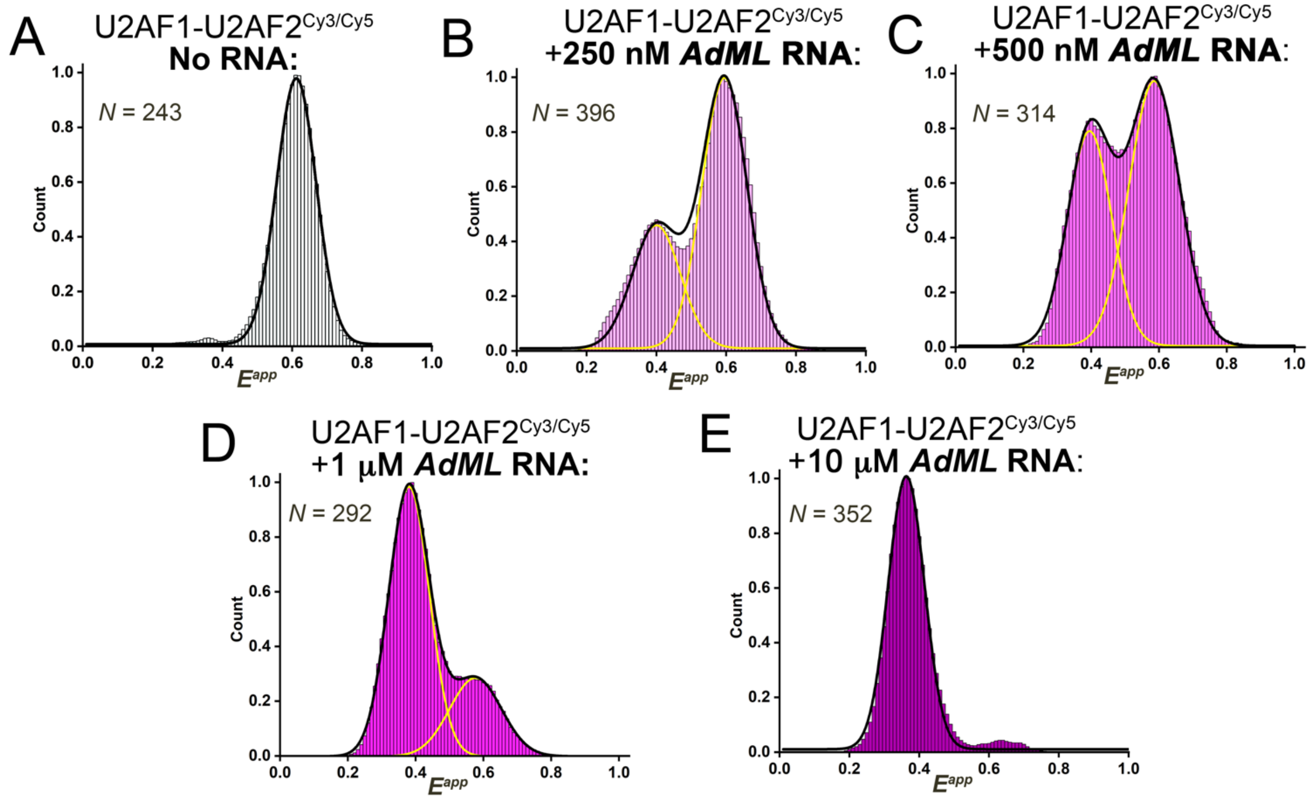

Supplementary Figure S4. Titration of tethered U2AF1–U2AF2$^{Cy3/Cy5}$ heterodimer with the indicated amounts of *AdML* splice site RNA increases the population of U2AF2$^{Cy3/Cy5}$ to a lower FRET state. Black or yellow lines indicate the respective summed or individual Gaussian fits of the histograms.

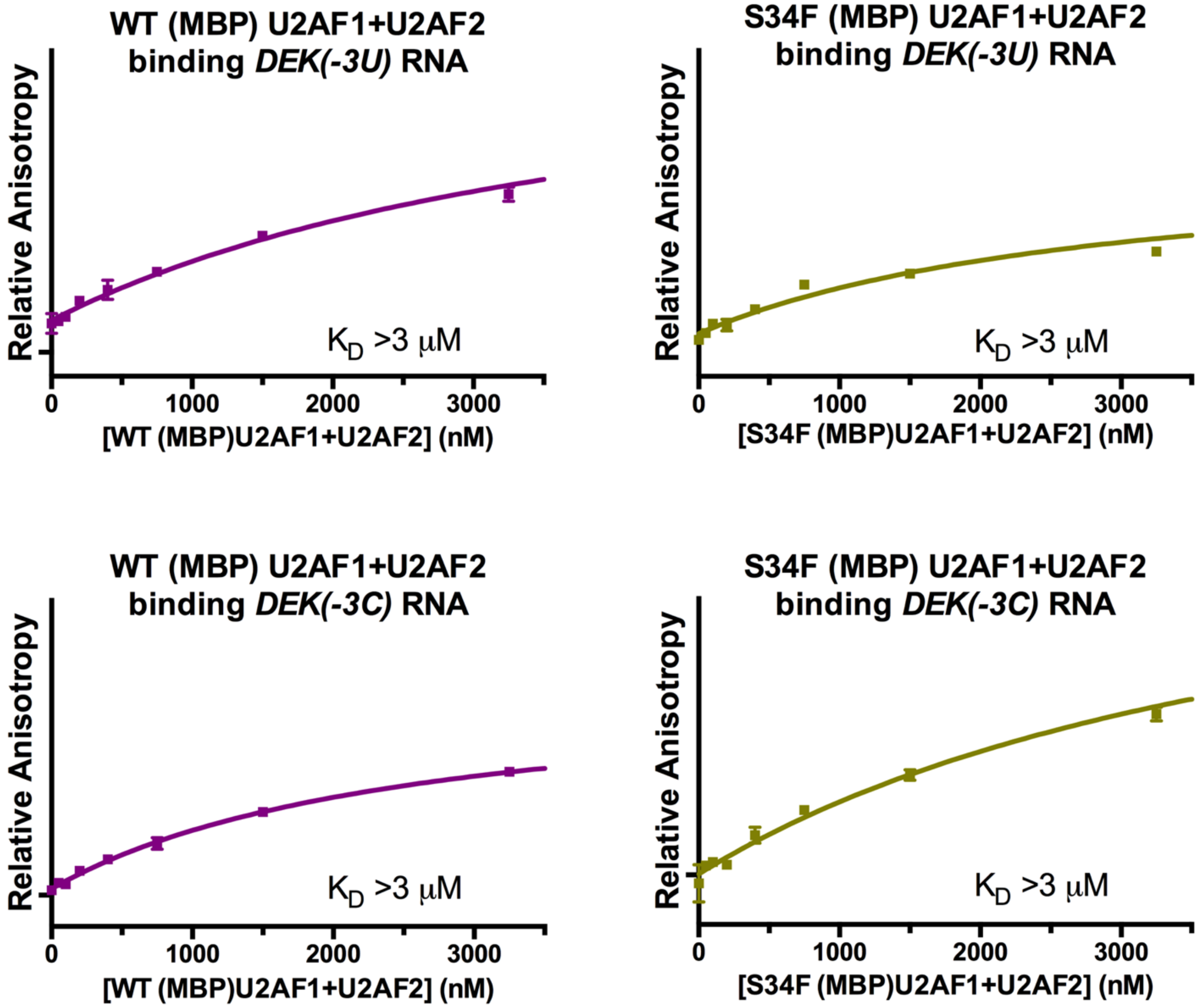

Supplementary Figure S5. Representative fluorescence anisotropy curves for human MBP-tagged U2AF1+U2AF2 heterodimer binding “weak” *DEK(-3U)* or *DEK(-3C)* splice site RNAs. The RNA oligonucleotide sequences are identical to those tested with the untagged SF1+U2AF1+U2AF2 ternary complex in Okeyo-Owuor *et al.* (2015) *Leukemia*, **29**, 909-917. The apparent equilibrium dissociation constants (K_D) are beyond the limits of the accessible protein concentrations due to the low binding affinities of the heterodimer for these non-consensus splice sites.
